# Identification of Novel Biomarkers for Predicting Prognosis and Immunotherapy Response in Head and Neck Squamous Cell Carcinoma Based on ceRNA Network and Immune Infiltration Analysis

**DOI:** 10.1155/2021/4532438

**Published:** 2021-12-06

**Authors:** Ya Guo, Wei Kang Pan, Zhong Wei Wang, Wang Hui Su, Kun Xu, Hui Jia, Jing Chen

**Affiliations:** ^1^Department of Radiation Oncology, The Second Affiliated Hospital, Xi'an Jiao Tong University, Xi'an, 710004 Shaanxi, China; ^2^Department of Pediatric Surgery, The Second Affiliated Hospital, Xi'an Jiao Tong University, Xi'an, 710004 Shaanxi, China

## Abstract

**Objectives:**

Patients with head and neck squamous cell carcinoma (HNSCC) have poor prognosis and show poor responses to immune checkpoint (IC) inhibitor (ICI) therapy. Competing endogenous RNA (ceRNA) networks, tumor-infiltrating immune cells (TIICs), and ICIs may influence tumor prognosis and response rates to ICI therapy. This study is aimed at identifying prognostic and IC-related biomarkers and key TIIC signatures to improve prognosis and ICI therapy response in HNSCC patients.

**Methods and Results:**

Ninety-five long noncoding RNAs (lncRNAs), microRNAs (miRNAs), and 1746 mRNAs were identified using three independent methods. We constructed a ceRNA network and estimated the proportions of 22 immune cell subtypes. Ten ceRNAs were related to prognosis according to Kaplan–Meier analysis. Two risk signatures based, respectively, on nine ceRNAs (ANLN, CFL2, ITGA5, KDELC1, KIF23, NFIA, PTX3, RELT, and TMC7) and three immune cell types (naïve B cells, neutrophils, and regulatory T cells) via univariate Cox regression, least absolute shrinkage and selection operator, and multivariate Cox regression analyses could accurately and independently predict the prognosis of HNSCC patients. Key mRNAs in the ceRNA network were significantly correlated with naïve B cells and regulatory T cells and with stage, grade, and immune and molecular subtype. Eight IC genes exhibited higher expression in tumor tissues and were correlated with eight key mRNAs in the ceRNA network in HNSCC patients with different HPV statuses according to coexpression and TIMER 2.0 analyses. Most drugs were effective in association with expression of these key signatures (ANLN, CFL2, ITGA5, KIF23, NFIA, PTX3, RELT, and TMC7) based on GSCALite analysis. The prognostic value of key biomarkers and associations between key ceRNAs and IC genes were validated using online databases. Eight key ceRNAs were confirmed to predict response to ICI in other cancers based on TIDE analysis.

**Conclusions:**

We constructed two risk signatures to accurately predict prognosis in HNSCC. Key IC-related signatures may be associated with response to ICI therapy. Combinations of ICIs with inhibitors of eight key mRNAs may improve survival outcomes of HNSCC patients.

## 1. Introduction

Head and neck cancer (HNC) is among the most common malignancies worldwide, accounting for about 600,000 new cases annually and 330,000 deaths [1]. Head and neck squamous cell carcinoma (HNSCC) is the predominant pathological subtype, comprising more than 90% of HNC cases [2]. Despite advances in surgery and radiotherapy, the 5-year overall survival (OS) rate for HNSCC is still unsatisfactory [3, 4]. Thus, there is an indisputably urgent need to identify effective biomarkers to predict prognosis and OS of patients with HNSCC.

Regulatory competing endogenous RNA (ceRNA) networks, consisting of long noncoding RNAs (lncRNAs), microRNAs (miRNAs), and mRNAs, have a crucial role in the processes of tumor occurrence and progression and in prediction of prognosis [5, 6]. Furthermore, accumulating evidence suggests that immune cell infiltration may have a significant impact on the prognosis of HNSCC patients [7, 8]. Although the prognostic value of ceRNA networks and immune cell infiltration in HNSCC have been reported, few studies have combined ceRNA networks and immune cell infiltration to predict prognosis in HNSCC patients; moreover, these studies have limitations, and their conclusions have been inconsistent. Further, few studies have had their results successfully confirmed in another independent database, and in many, a single analytical method was used to explore key molecules or immune cells [5, 9–11]. Moreover, in HNSCC, multiple clinical characteristics, including human papillomavirus (HPV) status, immune subtype, molecular subtype, grade, and stage, are associated with prognosis [12, 13]. There has not been sufficient systematic evaluation of the association among ceRNA-network RNAs, immune cell infiltration, and these clinical characteristics to fully elucidate the roles of these factors in prognosis. Therefore, a systematic scientific approach is needed to identify effective biomarkers for risk assessment of patient prognosis.

HNSCCs comprise a group of highly heterogeneous and immunosuppressive cancers.

Immunotherapy is aimed at increasing the activity of the immune system to eliminate cancer cells [14]. Immune checkpoint (IC) inhibitors (ICIs) represent a broadly effective immunotherapy that can block inhibitory IC pathways to restore antitumor immunity.

Anti-PD1/PDL1 ICIs can improve antitumor immune activity [4]. Although ICIs exert beneficial therapeutic effects on HNSCC, the response rate to them is still low [15]. Therefore, identifying biomarkers able to predict ICI treatment response can contribute to patient screening and individualized treatment approaches and is of great significance for standardizing immunosuppressive therapy and improving the prognosis of HNSCC patients. Previous studies have revealed that the expression of immunosuppressive molecules and tumor-infiltrating immune cells (TIICs) may regulate the response rate to immunotherapy and prognosis in HNSCC patients. A previous study proposed the epithelial–mesenchymal transition CYT Index as a superior predictor of prognosis and immunotherapy response across HNSCC [16], and immune cell infiltration scores were identified as sensitive prognostic biomarkers and predictive indicators for immunotherapy response [8]. However, no reliable biomarker for predicting response to ICIs and prognosis in HNSCC has yet been identified [17, 18].

In this study, mRNA, lncRNA, and miRNA expression profiles of HNSCC were downloaded from The Cancer Genome Atlas (TCGA) database to construct a ceRNA network. CIBERSORT (cell type identification by estimating relative subset of known RNA transcripts) was used to estimate the abundance of 22 immune cells based on TCGA dataset. Furthermore, two prognostic signatures were developed using a comprehensive bioinformatics method. Next, the association between key genes and immune cells was evaluated via coexpression analysis. We systematically investigated the associations between key ceRNA signatures and clinical characteristic (including stage, grade, and immune and molecular subtype). Moreover, IC correlation analysis and key ceRNA-related drug targets were performed to assess the predictive ability of these biomarkers with respect to ICI treatment response in HNSCC and to explore potential targets for improving the effectiveness of immunotherapy. Finally, the prognostic value of key biomarkers and the associations between key ceRNAs and IC genes were validated using online databases.

## 2. Materials and Methods

### 2.1. Data Analysis and ceRNA Network Construction

RNA sequencing (RNA-seq) and miRNA-seq data and clinical information for HNSCC patients were extracted from TCGA database (https://gdc-portal.nci.nih.gov/) [19]. HTseq-count profiles were obtained. In addition, practical extraction and reports Language (Perl) scripts were used to merge all clinical data. The R software (version 3.6.3, https://www.r-project.org/) was used to process downloaded files, to convert and eliminate unqualified data, to apply a relatively conservative approach to the data analysis, and to identify a suitable number of differentially expressed mRNAs (DEmRNAs), miRNAs (DEmiRNAs), and lncRNAs (DElncRNAs). The limma, edgeR, and DESeq2 R packages were used for differential expression analysis. Log fold change > 1 or <−1 and false discovery rate- (FDR-) adjusted *P* value < 0.05 were used as the thresholds to identify significantly expressed biomarkers. Only biomarkers that were identified using three independent analysis methods were selected as the final DEmRNAs, DEmiRNAs, and DElncRNAs [20]. A ceRNA network was established using the “GDCRNATools” R package. The ceRNA network was evaluated by hypergeometric testing and correlation analysis. *P* < 0.05 was considered to indicate a statistically significant result [21]. Finally, Cytoscape v.3.7.2 was used to visualize the ceRNA network [22].

### 2.2. Construction and Confirmation of ceRNA-Related Prognostic Model

We performed univariate Cox regression, least absolute shrinkage and selection operator (LASSO), and multivariate Cox regression analyses to identify prognosis-related signatures. Moreover, we plotted receiver operating characteristic (ROC) curves for 1-year, 3-year, and 5-year survival and calculated the corresponding area under the curve (AUC) values using the survival and time ROC packages to assess the predictive ability of the ceRNA-related signatures [23]. An AUC of <0.5 was considered insignificant. Patients were then divided into high- and low-risk groups according to the average risk score. We further calculated survival differences between the high- and low-risk groups [24]. Next, we performed univariate and multivariate Cox regression analyses to determine whether the risk score based on key ceRNAs was independent of other clinical characteristics (age, sex, grade, and stage) in the prediction of HNSCC prognosis. A hazard ratio (HR) > 1 and *P* < 0.05 were considered to indicate unfavorable prognostic factors, whereas HR < 1 and *P* < 0.05 indicated favorable prognostic factors [25].

### 2.3. Construction of Immune Cell-Related Prognostic Model

The CIBERSORT algorithm was used to estimate the abundance of 22 immune cell subtypes according to the RNA-seq count data [26]. The associations between immune cell types and prognosis were investigated by univariate Cox regression, LASSO regression, and multivariate Cox regression. We further assessed the sensitivity and specificity of immune cell-related prognostic models using ROC curves. In addition, we performed an independent prognostic analysis to assess whether the risk score based on key immune cells could predict HNSCC prognosis independently of other clinical characteristics.

### 2.4. Associations between Key ceRNAs and Significant Immune Cell Subtypes

The associations between significant mRNAs in the ceRNA network and key immune cells were investigated using Pearson correlation coefficients. Moreover, we analyzed the associations between the gene risk score and key immune cells.

### 2.5. Clinical Correlation Analysis

The Tumor Immune System Interactions Database (TISIDB; http://cis.hku.hk/TISIDB) was used to explore Spearman correlations between the identified key ceRNAs and clinical characteristics. Moreover, the expression distribution patterns of nine key ceRNAs across immune and molecular subtypes were determined using TISIDB [27]. The immune subtypes comprised five groups: C1 (wound healing), C2 (IFN-gamma dominant), C3 (inflammatory), C4 (lymphocyte depleted), and C6 (TGF-*β* dominant) [12]. HNSCC can be classified into four molecular subtypes, namely, atypical (AT), basal (BA), classical (CL), and mesenchymal (MS) [13].

### 2.6. Correlations between Immunotherapy Response and Key ceRNAs

The expression of ICs is correlated with the immune response to immunotherapy. We extracted the expression levels of eight immune-checkpoint genes (CD274, CTLA4, HAVCR2, LAG3, PDCD1, PDCD1LG2, TIGIT, and SIGLEC15) and compared their differences in expression between tumor and normal tissues in HNSCC using the R packages limma, ggplot2, and pheatmap. Coexpression analysis was used to define the correlations between the expression levels of nine key ceRNAs and those of the eight immune-checkpoint genes. A two-gene correlation map was obtained using the R package ggstatsplot. A *P* value of less than 0.05 was considered to indicate statistical significance. The Tumor Immune Dysfunction and Exclusion (TIDE, http://tide.dfci.harvard.edu) algorithm was then used to estimate the predictive power of the custom biomarker with respect to response outcome and OS [28]. We confirmed the correlations between the eight key mRNAs and response to ICIs in other cancers using TIDE.

### 2.7. Correlations between Expression of Key ceRNAs and Drug Sensitivity

Gene Set Cancer Analysis (GSCALite, http://bioinfo.life.hust.edu.cn/web/GSCALite/) is a web server for dynamic analysis and visualization of gene sets in cancer and drug sensitivity correlations. The expression of each gene in the gene set was determined, and small molecule/drug sensitivity (half-maximal inhibitory concentration; IC50) was obtained using Spearman's correlation analysis. Correlations with FDR < 0.05 were considered to represent results [29–31]. We used GSCALite to analyze the correlations between drug sensitivity and the signature of nine key ceRNAs.

### 2.8. Validation of Results

LOGpc (Long-term Outcome and Gene Expression Profiling Database of pan-cancers, http://bioinfo.henu.edu.cn/DatabaseList.jsp) includes 209 gene expression datasets and provides 13 types of survival terms for 31,310 patients with 27 distinct malignancies [32]. We first confirmed the prognostic value of major target genes using LOGpc datasets. Next, we confirmed the prognostic value of key immune cell types and ceRNAs using the TIMER 2.0 database [33]. Finally, we confirmed the expression of eight immune-checkpoint genes and determined the correlations between the expression levels of these genes and nine key genes in HNSCC with different HPV statuses using the gene correlation TIMER 2.0 module (http://timer.cistrome.org/) [34].

### 2.9. Statistical Analysis

All statistical analyses were carried out using R (version 3.6.3, https://www.r-project.org/) and the limma, GDCRNATools, ggplot2, rms, glmnet, survminer, and timeROC packages. A two-sided *P* value of less than 0.05 was considered to indicate statistical significance.

## 3. Results

### 3.1. Identification of Differentially Expressed Genes, DEmiRNAs, and DElncRNAs

**Figure 1:**
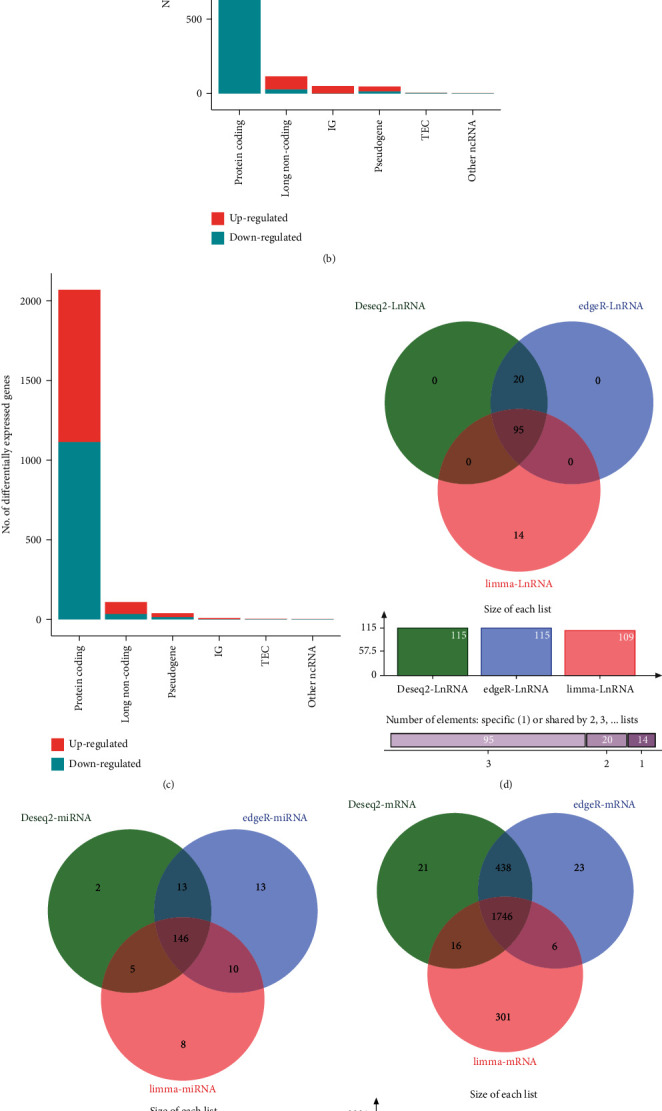
Identification of DElncRNAs, DEmiRNAs, DEmRNAs, and construction of the ceRNA network. Differentially expressed genes were identified by three independent methods. (a) Deseq2, (b) edgeR, and (c) limma. (d–f) Venn diagram displaying the intersection of (d) DElncRNAs, (e) DEmiRNAs, and (f) DEmRNAs. (g) Construction of the ceRNA network. Red circles represent miRNAs, green circles represent lncRNAs, and purple circles represent mRNAs. TEC: to be experimentally confirmed; IG: immunoglobulin. Log fold change > 1 or <−1 and FDR-adjusted *P* value < 0.05 were used to identify significantly expressed biomarkers.

We identified 115 DElncRNAs, 166 DEmiRNAs, and 2221 DEmRNAs using the DESeq2 package in the R programming language. Of these, 115 lncRNAs, 182 miRNAs, and 2213 mRNAs were found to be differentially expressed in HNSCC using the edgeR method. Next, we acquired 109 DElncRNAs, 169 DEmiRNAs, and 2069 DEmRNAs using the R package limma. A total of 95 DElncRNAs, 146 DEmiRNAs, and 1746 DEmRNAs were identified using three independent methods (Figures 1(a)–1(f)).

### 3.2. Construction of ceRNA Network and Survival Analysis

**Figure 2:**
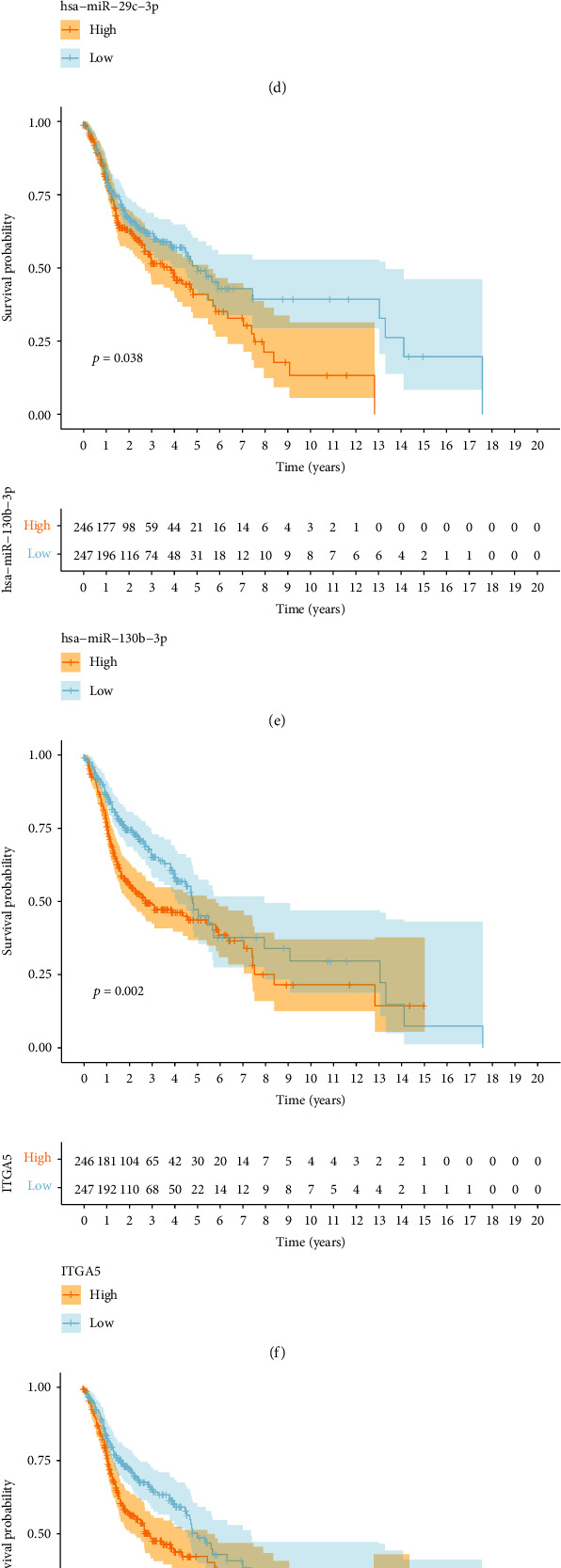
Survival analysis of key members in the ceRNA network. (a–j) K-M survival curves for the significant members of the ceRNA network. (a) ACSL1, (b) CDCA4, (c) GNA12, (d) hsa-miR-29c-3p, (e) hsa-miR-130b-3p, (f) ITGA5, (g) KDELC1, (h) PRKAA2, (i) PRUNE2, and (j) PTX3.

We constructed a lncRNA–miRNA–mRNA ceRNA network, which included three lncRNAs, eight miRNAs, and 69 mRNAs (Figure 1(g)). Then, Kaplan–Meier (K-M) survival analysis was performed to identify prognostic members in the constructed ceRNA network. Our results showed that 10 key markers, including ACSL1, CDCA4, GNA12, hsa-miR-29c-3p, hsa-miR-130b-3p, ITGA5, KDELC1, PRKAA2, PRUNE2, and PTX3, were significantly correlated with survival (Figures 2(a)–2(j)).

### 3.3. Construction of ceRNA-Related Prognostic Model

**Figure 3:**
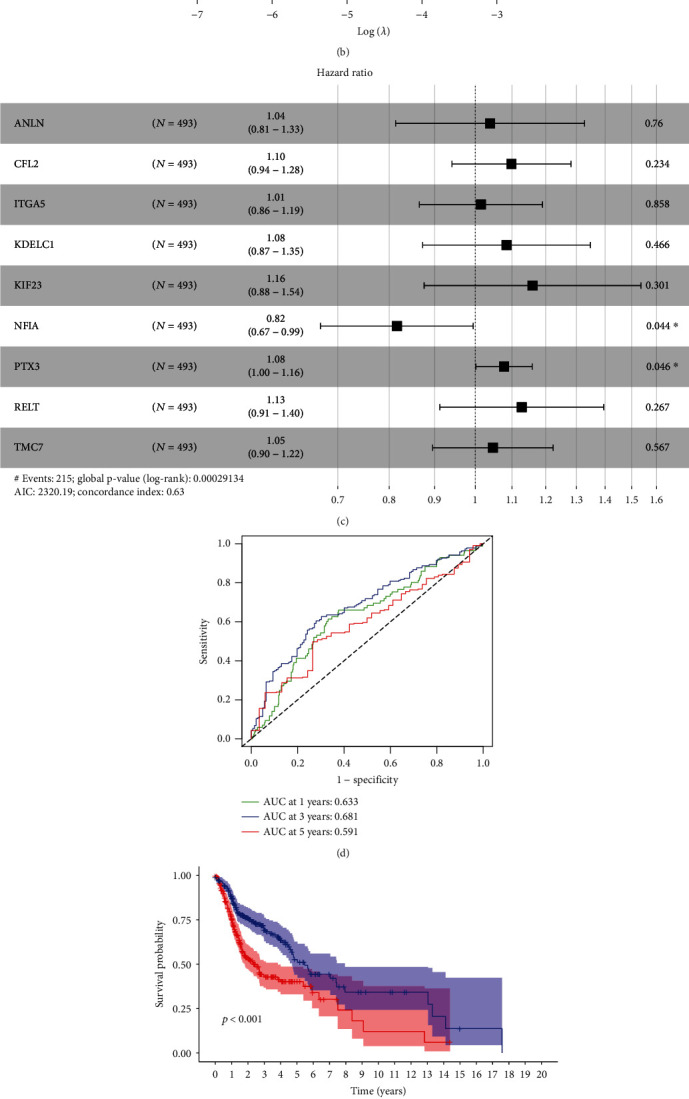
Development and validation of the ceRNA-related prognostic model. (a–c) LASSO and multivariate Cox regression analyses were applied to investigate the correlation between OS and ceRNAs. (d) ROC analysis showing the accuracy of the prediction model. (e) K-M survival curves comparing the high-risk and low-risk groups and the demonstrating predictive ability of our model. (f, g) Assessment of the independence of ceRNA-related prognostic model through (f) univariate and (g) multivariate Cox regression analyses. HR > 1 and *P* value < 0.05 indicate poor prognostic factors. HR < 1 and *P* values < 0.05 indicate favorable prognostic factors.

Thirteen ceRNAs were identified as prognosis-related signatures using univariate Cox regression (Table 1). LASSO regression analysis revealed that nine key markers were essential for modeling (Figures 3(a) and 3(b)); these were subjected to multivariable model analysis. Only two key biomarkers (NFIA and PTX3) had a significant impact on HNSCC prognosis (Figure 3(c)). In addition, analysis of the ROC curves indicated that the AUCs for 1-, 3-, and 5-year OS were 0.633, 0.681, and 0.591, respectively (Figure 3(d)). We further plotted survival curves for the high- and low-risk groups. As shown in Figure 3(e), patients in the low-risk group had significantly longer OS probability than those in the high-risk group. Finally, univariate and multivariate Cox regression analyses demonstrated that the risk score based on key ceRNAs was an independent predictor of poor prognosis in HNSCC patients (Figures 3(f) and 3(g)).

### 3.4. Immune Cell Infiltration Analysis

**Figure 4:**
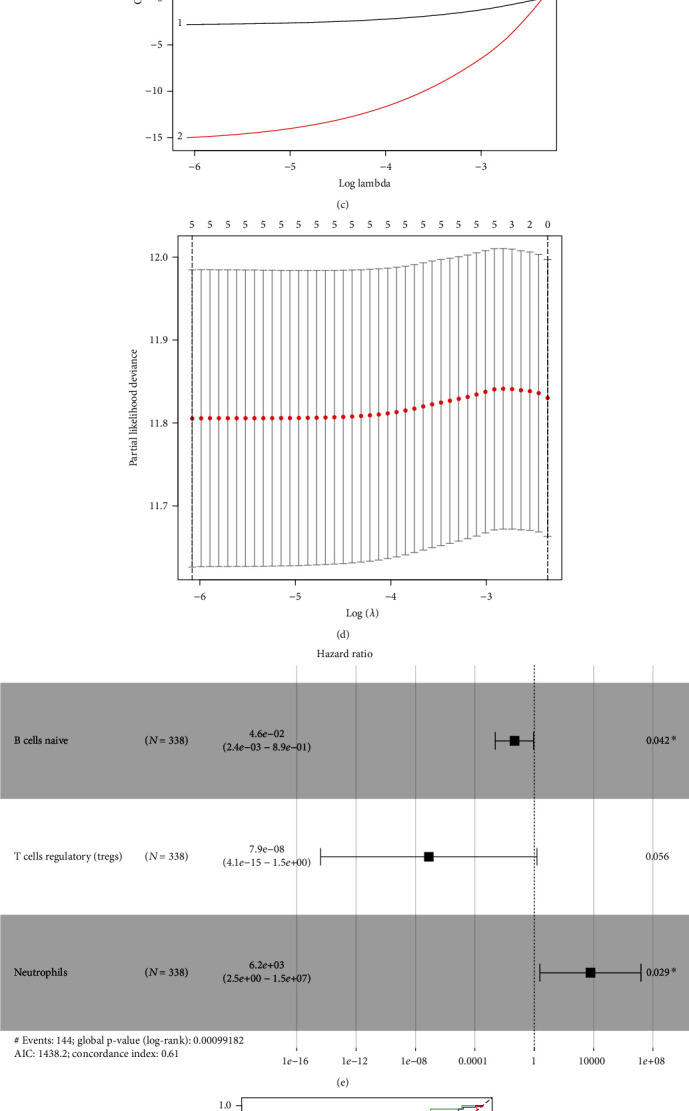
Construction and validation of the immune cells related prognostic model. (a) Distribution of 22 immune cell subtypes estimated by CIBERSORT in HNSCC. (b) Heatmap showing the levels of 22 immune cell subtypes in tumor and normal samples. (c–e) LASSO and multivariate Cox regression were used to investigate the correlation between OS and immune cell infiltration. (f, g) ROC and Kaplan–Meier survival curves showing the accuracy of immune cell subtypes and related prognostic models for predicting OS. (h) Univariate analysis and (i) multivariate Cox regression analysis confirmed the independence of the immune cell-related prognostic model.

CIBERSORT was used to assess immune cell infiltration in each HNSCC sample. The results revealed significant differences in the proportions of immune cell infiltration (Figure 4(a)). In addition, the generated heatmap showed that 11 immune cell subtypes, M1 macrophages, CD8 T cells, M0 macrophages, M2 macrophages, resting CD4 memory T cells, memory B cell, naïve B cells, and regulatory T cells (Treg), monocytes, resting myeloid dendritic cells, and activated mast cells presented significant different proportions between the tumor group and the normal group (Figure 4(b)).

### 3.5. Construction and Validation of Immune Cell-Related Prognostic Model

Univariate Cox regression, LASSO regression, multivariate Cox regression, and independent prognostic analysis were used to analyze the associations between different immune cell subtypes and prognosis. The results showed that three variables were related to prognosis in HNSCC: high infiltration of Tregs and naïve B cells was associated with a favorable prognosis, whereas increased levels of neutrophils were correlated with worse prognosis (Figures 4(c)–4(e)). According to ROC curve analysis, the AUCs of the 1-, 3-, and 5-year prognosis models were 0.625, 0.626, and 0.568, respectively (Figure 4(f)). The K-M curve analysis indicated that the high-risk group had an unfavorable prognosis (Figure 4(g)). Finally, we found that the immune cell-related risk score was an independent factor for predicting prognosis in HNSCC (Figures 4(h) and 4(i)).

### 3.6. Relationships between Key ceRNAs and Significant Immune Cell Signatures

**Figure 5:**
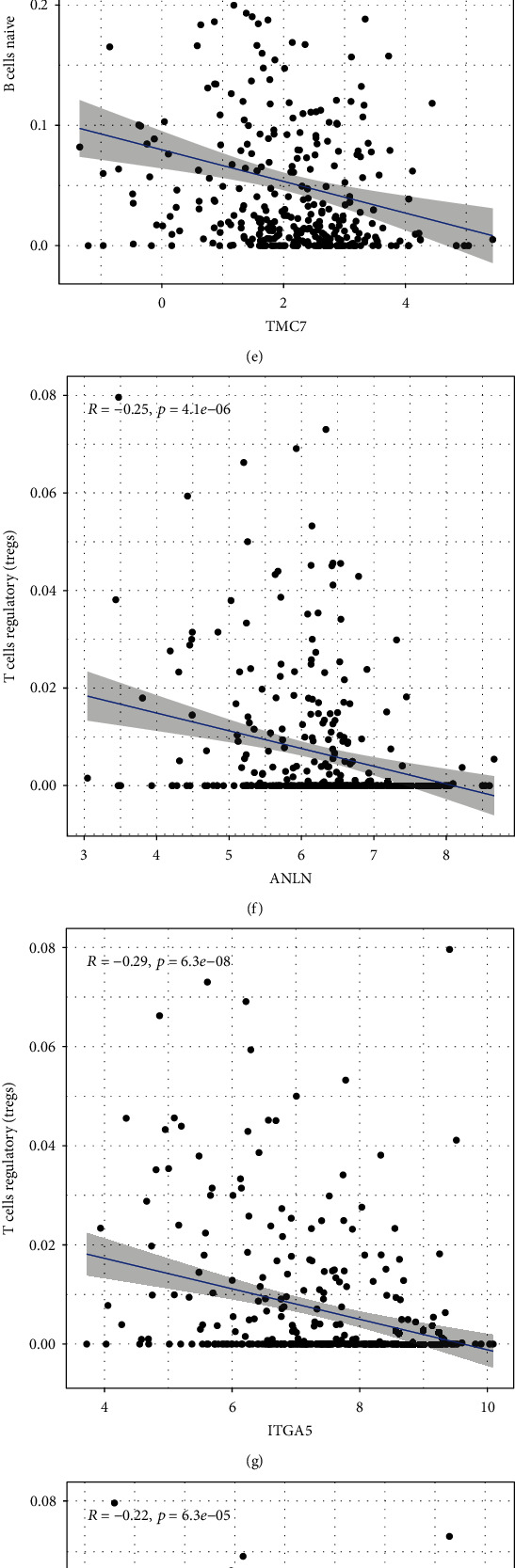
Analysis of the correlations of significant genes in the ceRNA network and gene risk score with immune cell infiltration. (a) Heatmap displaying correlations between prognostic immune cells and key genes in the ceRNA network. (b–e) Expression of CFL2, ITGA5, KDELC1, and TMC7 was negatively associated with infiltration of naïve B cells. (f–i) ANLN, ITGA5, KIF23, and TMC7 expression was negatively associated with Tregs. (j–l) Pearson's correlation analysis was performed to evaluate the associations between key immune cells and gene risk score.

There were significant correlations between key molecules in the ceRNA network and immune cell signatures (Figure 5(a)). The coexpression analysis results indicated that CFL2, ITGA5, KDELC1, and TMC7 were negatively associated with levels of naïve B cell infiltration (Figures 5(b)–5(e)), whereas ANLN, ITGA5, KIF23, and TMC7 expression was negatively associated with Treg levels (Figures 5(f)–5(i)). As shown in Figures 5(j)–5(l), significant immune cell signatures in different risk score exhibited statistical significance, and higher levels of naïve B cells were associated with lower risk scores (*P* = 0.0085). A similar result was observed for Tregs (*P* = 4*e* − 9).

### 3.7. Clinical Correlation Analysis

**Figure 6:**
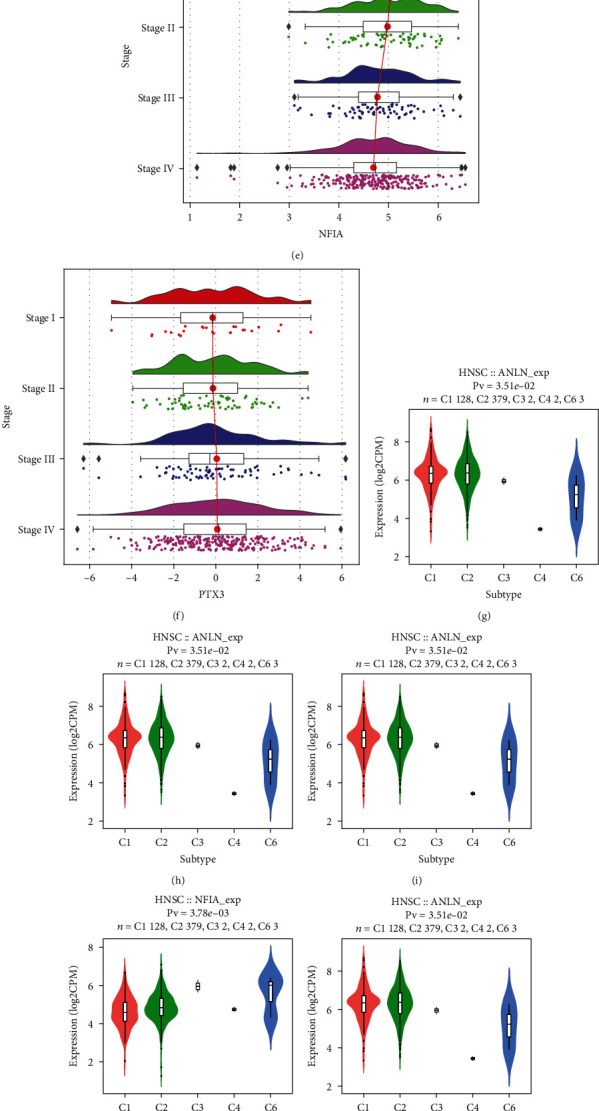
Clinical correlation analysis. (a, b) ANLN and KIF23 expression was associated with tumor grade according to TISIDB datasets. (a) ANLN and (b) KIF23. (c–f) Key mRNAs among ceRNAs related to tumor stage according to TISIDB. (c) ITGA5, (d) KDELC1, (e) NFIA, and (f) PTX3. (g–i) Distribution of the expression of key ceRNAs across immune subtypes according to TISIDB: (g) ANLN, (h) KDELC1, (i) KIF23, and (j) NFIA. (k–q) Distribution of key ceRNA expression across molecular subtypes (TISIDB): (k) CFL2, (l) ITGA5, (m) KDELC1, (n) NFIA, (o) PTX3, (p) RELT, and (q) TMC7. Statistical significance of differential expression evaluated using Kruskal–Wallis test. C1 (wound healing), C2 (IFN-gamma dominant), C3 (inflammatory), C4 (lymphocyte depleted), and C6 (TGF-*β* dominant). Molecular subtypes include four types, namely, atypical (AT), basal (BA), classical (CL), and mesenchymal (MS) based on biological characteristics of genes highly expressed in each subtype.

We evaluated the correlations of the nine key signatures with tumor grade and stage. Our results showed that increased expression of ANLN and KIF23 was associated with higher tumor grade (Figures 6(a) and 6(b)). Moreover, the expression levels of ITGA5, KDELC1, and PTX3 were positively correlated with the stage of HNSCC, whereas NFIA expression decreased at higher HNSCC stages (Figures 6(c)–6(f)). Furthermore, our results showed that ANLN, KDELC1, KIF23, and NFIA expression was associated with different immune subtypes. ANLN and KIF23 showed increased expression in subtypes C1 and C2. KDELC1 showed higher expression in the C1, C2, and C6 subtypes, indicating that it may be mainly associated with worse survival. NFIA showed higher expression in subtype C3 and decreased expression in C4, which predicted better survival (Figures 6(g)–6(i)). CFL2, ITGA5, KDELC1, and PTX3 were highly expressed in the MS subtype. Increased expression of RLET and TMC7 was mainly found in the basal subtype, and higher expression of NFIA was associated with the AT subtype (Figures 6(k)–6(q)).

### 3.8. Key ceRNA Expression Was Correlated with Immunotherapy Response and Drug Sensitivity

**Figure 7:**
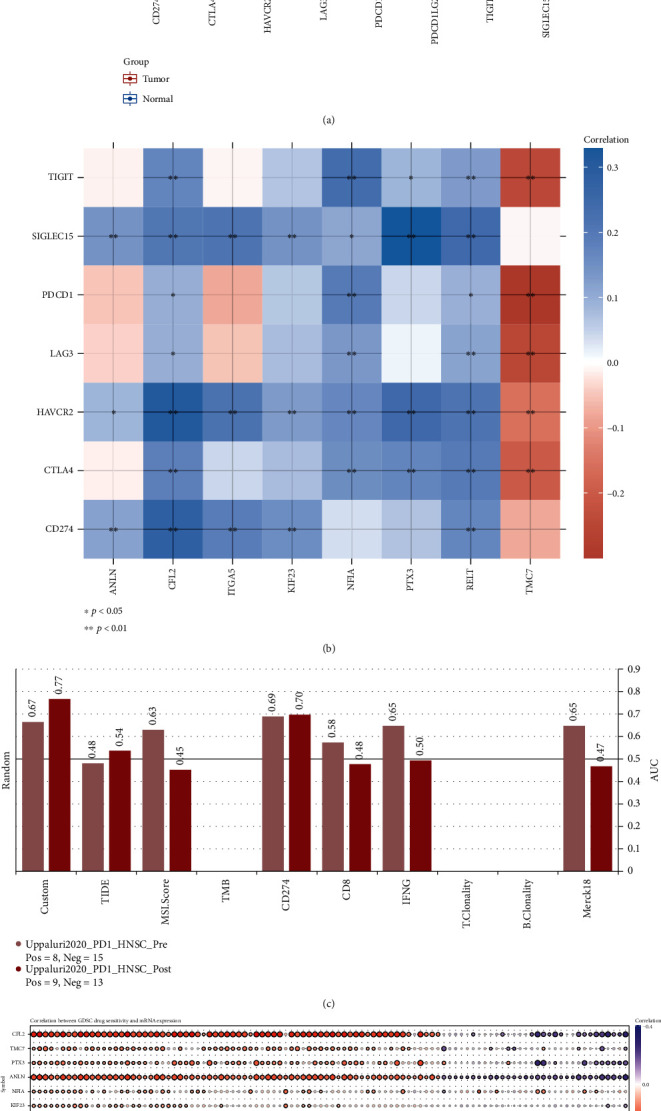
Key ceRNA expression correlates with immunotherapy response and drug sensitivity. (a) Differences in expression of tumor-related immunosuppressive molecules in HNSCC; ^∗^*P* < 0.05, ^∗∗^*P* < 0.01, and ^∗∗∗^*P* < 0.001. (b) Correlations between key ceRNAs and IC molecules. The horizontal and vertical coordinates represent genes. Different colors represent correlation coefficients (blue, positive correlation and red, negative correlation); the darker the color, the stronger the correlation; ^∗^*P* < 0.05 and ^∗∗^*P* < 0.01. (c) Comparison of AUC values between the custom biomarker and other published biomarkers in predicting anti-PD1 response. (d) Correlation analysis of drug sensitivity and expression of key ceRNAs.

Multiple IC genes including CD274, CTLA4, HAVCR2, LAG3, PDCD1, SIGLEC15, TIGIT, and TIM3 exhibited higher expression in tumor tissues compared with normal tissues (Figure 7(a)). The expression of ANLN, ITGA5, and KIF23 was positively associated with the expression of CD274 (PDL1), HAVCR2, and SIGLEC15. CFL2 and RELT expression was positively correlated with the expression of CD274 (PDL1), CTLA4, HAVCR2, LAG3, PDCD1 (PD1), SIGLEC15, and TIGIT. NFIA was positively related to the abovementioned seven immunosuppressive genes, except for CD274. Overexpression of PTX3 was associated with increased CTLA4, HAVCR2, SIGLEC15, and TIGIT expression. TMC7 expression was negatively correlated with the expression of CTLA4, HAVCR2, LAG3, PDCD1 (PD1), and TIGIT (Figure 7(b)).

In addition, our biomarker could effectively predict anti-PD1 response compared with published biomarkers (MSI, CD274, CD8, IFNG, etc.) (Figure 7(c)). Moreover, Spearman's correlation analysis was performed to explore the correlations of the expression of the nine key genes with drug sensitivity in terms of IC50 values. Our results showed that most drugs were effective in association with increased expression of CFL2, TMC7, PTX3, ANLN, NFIA, and KIF23, whereas RELT and ITGA5 were negatively regulated by most drugs. Specifically, these molecules could be exploited as potential therapeutic drug targets for HNSCC (Figure 7(d)).

### 3.9. Validation of the Prognostic Value of Biomarkers and Associations between Key ceRNAs and IC Genes

**Figure 8:**
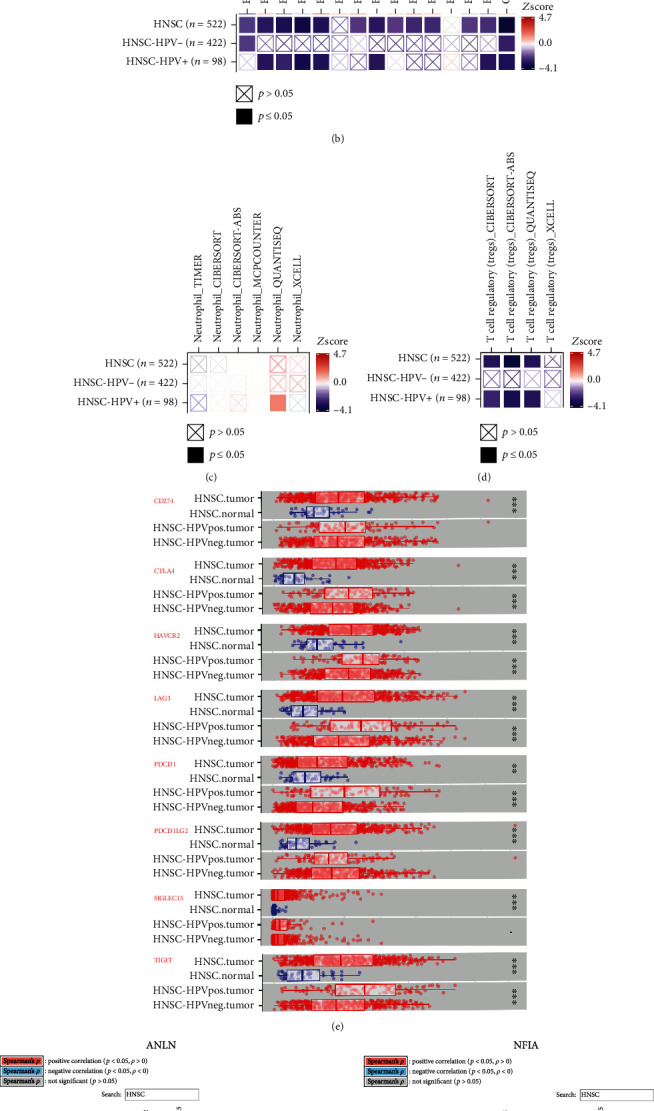
Validation of the prognostic value of identified biomarkers and associations between key ceRNAs and IC genes. (a) Confirmation of prognostic value of key ceRNAs in HNSCC with different HPV statuses via the TIMER 2.0 database. (b–d) Analysis of associations between key immune cells and OS in HNSCC patients with different HPV statuses based on Cox regression analysis using the TIMER 2.0 database (http://timer.cistrome.org/). (e) Differences in expression of IC genes with different HPV statuses in HNSCC using TIMER 2.0. (f) Exploration of the associations between key ceRNAs and IC molecules with different HPV statuses in HNSCC using TIMER 2.0.

LOGpc was used to perform survival analysis for key ceRNAs. The results indicated that higher expression levels of CFL2, ITGA5, KDELC1, PTX3, and RELT were correlated with poor prognosis in HNSCC patients, whereas increased expression of NFIA was associated with longer OS. There was no correlation between ANLN, KIF23, or TMC7 expression and prognosis in HNSCC patients (Table 2). The associations between pivotal ceRNAs and prognosis were then determined using TIMER 2.0. We found that high expression levels of ANLN, CFL2, ITGA5, KDELC1, PTX3, RELT, and TMC7 were significantly associated with shorter OS in HNSCC patients, whereas higher expression of NFIA was correlated with longer OS. In HPV-positive HNSCC patients, high expression of ITGA5, KDELC1, and TMC7 was associated with poor prognosis, whereas high NFIA expression was correlated with good prognosis. ANLN, CFL2, KDELC1, PTX3, and RELT were associated with unfavorable prognosis in HPV-negative HNSCC (Figure 8(a)). In addition, the prognostic roles of immune cells in HNSCC patients with different HPV statuses were confirmed using the TIMER 2.0 database. In both HPV-positive and HPV-negative HNSCC patients, high infiltration levels of naïve B cells and Tregs were associated with a favorable prognosis according to several algorithms, whereas high neutrophil infiltration indicated an unfavorable prognosis (Figures 8(b)–8(d)). Eight tumor-related immunosuppressive molecules, CD274, CTLA4, HAVCR2, LAG3, PDCD1 (PD1), PDCD1LG2, SIGLEC15, and TIGIT, had higher expression in HNSCC tumor tissues compared with HNSCC normal tissues.

Notably, most IC molecules showed significantly increased expression in HPV-positive HNSCC patients (Figure 8(e)). We also found that the expression of these ICI factors was significantly positively correlated with the expression of seven key mRNAs (ANLN, CFL2, ITGA5, KIF23, NFIA, PTX3, and RELT), whereas the expression of immunosuppressive molecules was negatively associated with TMC7 expression, especially in HPV-positive HNSCC patients (Figure 8(f)). We further discovered that eight key ceRNAs were correlated with response to ICI in other cancers (melanoma, bladder, kidney, and glioblastoma) (Table 3).

These results are consistent with the results of our study.

## 4. Discussion

HNSCC is among the deadliest malignancies in humans and a significant cause of cancer-related deaths worldwide [35]. Despite advances in the screening, diagnosis, and treatment of HNSCC in recent decades, especially in terms of immunotherapy, the prognosis of HNSCC patients remains very poor [36]. Recent studies have explored the roles of immune cell infiltration and ceRNA networks in HNSCC. However, these studies have failed to accurately identify key molecules for predicting the prognosis of HNSCC [9, 10, 37]. Accumulating evidence suggests that machine learning is a reliable and robust technique that can be used to quickly and accurately identify critical biomarkers [38, 39]. ICIs may exert beneficial therapeutic effects in HNSCC, although the response rate to ICIs remains poor [15]. A lncRNA signature of tumor-infiltrating B lymphocytes was found to have potential applications in prognostic prediction and immunotherapy for bladder cancer based on computational recognition [40], and lncRNAs associated with tumor immune infiltration were identified as improving clinical outcomes and immunotherapy response in non-small-cell lung cancer based on comprehensive analysis [41]. Combinations of ceRNA with ICIs and TIICs for improving prognosis and immunotherapy response in HNSCC are limited. Therefore, it is crucial to establish predictive biomarkers for selecting patients who will be responsive to ICI therapy.

In the present study, we defined nine key ceRNAs (ANLN, CFL2, ITGA5, KDELC1, KIF23, NFIA, PTX3, RELT, and TMC7) and three immune cell subtypes (naïve B cells, Tregs, and neutrophils) using univariate Cox analysis, LASSO, multivariate Cox analysis, and independent prognostic analysis. Specifically, KDELC1, PTX3, and ITGA5 were found to be associated with poor prognosis in both the K-M survival analysis and the multivariate Cox analysis. ROC curve analysis was used to further evaluate both signatures and confirm their favorable predictive and prognostic abilities.

We identified a potential regulatory mechanism involving KCNQ1OT1 (lncRNA), miR-338-3p (miRNA)/miR-29c-3p, ITGA5 (mRNA)/KDELC1, and naïve B cells/Tregs.

A previous report indicated that KCNQ1OT1 was associated with cell proliferation, apoptosis, prognosis, invasion, and metastasis in various cancers [10, 42–44]. Further, KCNQ1OT1 facilitated invasion and inhibited apoptosis in oral squamous cell carcinoma by regulating the miR-185-5p/Rab14 axis [45]. miR-338-3p was downregulated in esophageal squamous cell carcinoma and could act as a tumor suppressor [46]. In addition, miR-338-3p was shown to inhibit colorectal carcinoma cell invasion and migration by targeting smoothened [47] and was associated with favorable prognosis in urothelial carcinoma of the bladder [48]. Inhibition of miR-29c-3p was found to be associated with poor prognosis in patients with laryngeal squamous cell carcinoma [49]. ITGA5 has essential roles in tumorigenesis, migration, and invasion in various cancer types [10]. Studies have shown that ITGA5 is highly expressed in HNSCC, where its high expression is significantly associated with poor survival [50]. Overexpression of PTX3 has been related to poor prognosis in pancreatic cancer and is linked to more advanced stages of the disease [51]. Evidence also suggests that PTX3 promotes metastasis of cervical cancer and EGF-induced metastasis of HNSCC through upregulation of MMP-2 and MMP-9 [52]. Our results are in accordance with these findings and suggest that the KCNQ1OT1/miR-338-3p/ITGA5 and KCNQ1OT1/miR-29c-3p/KDELC1 axes have important roles in determining the prognosis of HNSCC patients.

The associations among the nine key ceRNA signatures, three immune cell signatures, and different clinicopathological features was assessed. The results showed that ANLN, KIF23, and ITGA5 expression was significantly correlated with grade. Increased KDELC1 and PTX3 expression was related to advanced stage, whereas overexpression of NFIA was correlated with low tumor stage (Figures 6(a)–6(f)). A recent study showed that the C1 and C2 subtypes were enriched in HNSCC and were associated with less favorable outcomes. The C3 subtype had the most favorable prognosis, whereas the C4 and C6 subtypes had the worst prognoses [53]. In our study, we observed increased expression of ANLN and KIF23 in subtypes C1 and C2, whereas KDELC1 showed higher expression in the C1, C2, and C6 subtypes, indicating that it may be associated with worse survival. NFIA showed increased expression in subtype C3 and decreased expression in C4, which indicated that it could predict better survival (Figures 6(g)–6(j)).

Recently, Walter et al. [13] reported that the MS subtype was associated with distant metastases, and that treatment with EGFR inhibitors was less likely to be effective in the AT subtype. Our results indicated that CFL2, ITGA5, KDELC1, and PTX3 were highly expressed in the MS subtype. Higher NFIA expression was associated with the AT subtype (Figures 6(k)–6(q)). These studies further support our finding that NFIA expression was correlated with favorable outcomes whereas higher expression of the other ceRNAs was associated with unfavorable outcomes.

Previous studies have revealed that naïve B cells and Tregs are indicators of better survival, whereas neutrophils have been associated with HPV positivity and poorer outcomes [7, 10, 54]. Higher Treg infiltration was found to be correlated with better prognosis and longer survival in HPV-positive patients compared with HPV-negative HNSCC patients [55]. By contrast, naïve B cells could be correlated with tumorigenesis and progression of HNSCC [10]. Neutrophils induce tumor progression and promote tumor cell migration in HNSCC; therefore, neutrophil infiltration represents a risk factor. [56, 57]. Our integrated analyses showed that three immune cell subtypes (naive B cells, Tregs, and neutrophils) could be used as prognosis-related biomarkers in HNSCC. Naïve B cells and Tregs were associated with favorable prognosis, whereas neutrophils represented an unfavorable prognostic marker in HNSCC, especially in HPV-positive HNSCC patients (Figures 4(e) and 8(b)–8(d)). ROC analysis showed that the key immune cell-related model constructed in this study could predict the prognosis of patients with HNSCC. As shown in Figures 5(j)–5(l), higher levels of naïve B cells and Tregs were related to lower risk scores (*P* = 0.0085 and *P* = 4*e* − 9). These results further suggested that these immune cell types (naïve B cells, Tregs, and neutrophils) were related to prognosis. Our results are consistent with those of previous studies, and our immune cell-related model could be used as an independent factor to assess prognosis in HNSCC.

Finally, we found that eight IC genes exhibited higher expression in tumor tissues and were correlated with key ceRNAs in HNSCC with different HPV statuses (Figures 7(a) and 7(b) and 8(e) and 8(f)). Our identified biomarker could more effectively predict anti-PD1 response compared with published biomarkers (Figure 7(c)). Previous studies have reported that HPV-positive HNCs expressed high levels of multiple T cell exhaustion markers, including LAG3, PD1, TIGIT, and TIM3; the high expression of these markers was correlated with improved survival in HPV-positive HNC patients [58, 59]. Based on these studies and our findings, HNSCC may exhibit strong beneficial responses to immunotherapy and high expression levels of these key genes may contribute to predicting improved survival of HNSCC patients, especially in cases of HPV-positive HNSCC. We also observed that eight key genes were associated with treatment response to most drugs, suggesting the potential of these molecules as therapeutic drug targets in HNSCC (Figure 7(d)). These results provide a scientific rationale for potentially combining ICI therapy with inhibitors of the eight key genes identified in this study to improve treatment efficacy for HNSCC patients.

## 5. Conclusions

We identified nine key ceRNAs and three immune cell-related signatures as potential biomarkers for predicting the prognosis of HNSCC. In addition, the KCNQ1OT1 (lncRNA), miR-338-3p (miRNA)/miR-29c-3p, ITGA5 (mRNA)/KDELC1, and naïve B cell/Treg axes may be linked to prognosis of HNSCC. As key IC-related members of ceRNAs may be associated with response to ICI therapy, combining ICI with inhibitors of these eight key genes may contribute to improving treatment efficacy in HNSC patients. However, additional clinical data and experiments are required to confirm the prognostic value of these signatures and their potential associations with ICI immunotherapy outcomes in patients with HNSCC. We will perform further experiments to confirm the current research results.

## Figures and Tables

**Table 1:** Identification of prognosis-related ceRNAs by univariate Cox regression analysis.

Id	HR	HR.95L	HR.95H	P value
ANLN	1.2194	1.0487	1.4179	0.0099
C1QTNF6	1.1279	1.0083	1.2617	0.0354
CFL2	1.2049	1.0554	1.3756	0.0058
DCBLD2	1.1560	1.0149	1.3166	0.0290
GNA12	1.3001	1.0687	1.5816	0.0087
ITGA5	1.2156	1.0892	1.3567	0.0005
KDELC1	1.3339	1.1295	1.5753	0.0007
KIF23	1.2437	1.0289	1.5034	0.0242
NFIA	0.7776	0.6488	0.9319	0.0065
PRUNE2	1.0667	1.0078	1.1292	0.0260
PTX3	1.1174	1.0509	1.1882	0.0004
RELT	1.2274	1.0165	1.4822	0.0332
TMC7	1.1395	1.0046	1.2926	0.0422

**Table 2:** Survival analysis for major target ceRNAs using LOGpc.

Symbol	Dataset	HR	95% CI	*P* value	Prognostic
CFL2	TCGA	1.3608	1.0137-1.8268	0.0403	Poor
ITGA5	GSE31056	2.6804	1.3752-5.2245	0.0038	Poor
ITGA5	TCGA	1.4878	1.1162-1.9832	0.0067	Poor
KDELC1	TCGA	1.4946	1.1212-1.9922	0.0061	Poor
KDELC1	GSE31056	2.1694	1.0960-4.2940	0.0262	Poor
NFIA	GSE65858	0.4627	0.2648-0.8086	0.0068	Good
PTX3	GSE31056	3.9765	2.0435-7.7379	<0.0001	Poor
PTX3	TCGA	1.3605	1.0103-1.8320	0.0426	Poor
RELT	TCGA	1.3554	1.0113-1.8165	0.0418	Poor

**Table 3:** Associations between gene expression and therapy outcome in clinical studies of IC blockade.

Gene	Cohort	Cancer	Subtype	Survival	Risk.adj
ANLN	Riaz2017_PD1	Melanoma	Ipi_Prog	OS	2.025
	Riaz2017_PD1	Melanoma	Ipi_naive	PFS	-3.136
CFL2	Liu2019_PD1	Melanoma	Ipi_naive	OS	2.489
ITGA5	Mariathasan2018_PDL1	Bladder	mUC	OS	3.52
	Miao2018_ICB	Kidney	Clear	PFS	-3.173
KIF23	Riaz2017_PD1	Melanoma	Ipi_Prog	OS	2.802
NFIA	Mariathasan2018_PDL1	Bladder	mUC	OS	2.987
	Zhao2019_PD1	Glioblastoma	Pre	PFS	-2.336
PTX3	Riaz2017_PD1	Melanoma	Ipi_naive	OS	2.467
RELT	Mariathasan2018_PDL1	Bladder	mUC	OS	2.505
	Liu2019_PD1	Melanoma	Ipi_naive	PFS	-2.32
TMC7	Hugo2016_PD1	Melanoma		OS	2.27
	Lauss2017_ACT	Melanoma		OS	-2.528

## Data Availability

The gene expression RNA-sequencing data and clinical information of HNSCC in the current study were obtained from TCGA data portal (https://portal.gdc.cancer.gov/).

## Supplementary Materials

Supplementary 1 Additional file 1. Original source data: online web service.

Supplementary 2 Additional file 2. Original source data: related R software.
