# Supplementary material for: Identification of Novel Biomarkers for Predicting Prognosis and Immunotherapy Response in Head and Neck Squamous Cell Carcinoma Based on ceRNA Network and Immune Infiltration Analysis
Source: Biomed Res Int. 2021 Dec 6;2021:4532438. doi: 10.1155/2021/4532438 (PMC8670464; doi:10.1155/2021/4532438)
Supplement: Supplementary 1 — Additional file 1. Original source data: online web service. [file 4532438.f1.docx]

**Supplemetary 1 Original source data-Online web service**

TCGA database :https://gdc-portal.nci.nih.gov/

LOGpc : http://bioinfo.henu.edu.cn/DatabaseList.jsp

TISIDB; http://cis.hku.hk/TISIDB

TIMER 2.0 : http://timer.cistrome.org/

GSCALite: http://bioinfo.life.hust.edu.cn/web/GSCALite/

R : version 3.6.3, https://www.r-project.org/
