# Supplementary material for: Identification of Novel Biomarkers for Predicting Prognosis and Immunotherapy Response in Head and Neck Squamous Cell Carcinoma Based on ceRNA Network and Immune Infiltration Analysis
Source: Biomed Res Int. 2021 Dec 6;2021:4532438. doi: 10.1155/2021/4532438 (PMC8670464; doi:10.1155/2021/4532438)
Supplement: Supplementary 2 — Additional file 2. Original source data: related R software. [file 4532438.f2.docx]

**Supplemetary 2 Original source data-Related R software**

1. Deseq2-GDCRNATools

#if (!requireNamespace("BiocManager", quietly = TRUE))

# install.packages("BiocManager")

#BiocManager::install("GDCRNATools")

#install.packages("ggplot2")

library(GDCRNATools)

library(ggplot2)

adjpFilter=0.05

logFCfilter=1

FCfilter=2^logFCfilter

hyperPfilter=0.05

corPfilter=0.05 setwd("C:\\Users\\86186\\Desktop\\11.GDCRNATools")

project <- 'TCGA-HNSC'

rnadir <- paste(project, 'RNAseq', sep='/')

mirdir <- paste(project, 'miRNAs', sep='/')

gdcRNADownload(project.id = project,

data.type = 'RNAseq',

write.manifest = FALSE,

method = 'gdc-client',

directory = rnadir)

gdcRNADownload(project.id = project,

data.type = 'miRNAs',

write.manifest = FALSE,

method = 'gdc-client',

directory = mirdir)

metaMatrix.RNA <- gdcParseMetadata(project.id = project,

data.type = 'RNAseq',

write.meta = FALSE)

metaMatrix.RNA <- gdcFilterDuplicate(metaMatrix.RNA)

metaMatrix.RNA <- gdcFilterSampleType(metaMatrix.RNA)

metaMatrix.MIR <- gdcParseMetadata(project.id = project,

data.type = 'miRNAs',

write.meta = FALSE)

#miRNA metadata

metaMatrix.MIR <- gdcFilterDuplicate(metaMatrix.MIR)

metaMatrix.MIR <- gdcFilterSampleType(metaMatrix.MIR)

rnaCounts <- gdcRNAMerge(metadata = metaMatrix.RNA,

path = rnadir,

organized = FALSE, ## if target data are in folders

data.type = 'RNAseq')

mirCounts <- gdcRNAMerge(metadata = metaMatrix.MIR,

path = mirdir,

organized = FALSE, ## if target data are in folders

data.type = 'miRNAs')

rnaExpr <- gdcVoomNormalization(counts = rnaCounts, filter = FALSE)

mirExpr <- gdcVoomNormalization(counts = mirCounts, filter = FALSE)

DEGAll <- gdcDEAnalysis(counts = rnaCounts,

group = metaMatrix.RNA$sample_type,

comparison = 'PrimaryTumor-SolidTissueNormal',

method = 'DESeq2')

degMI <- gdcDEAnalysis(counts = mirCounts,

group = metaMatrix.MIR$sample_type,

comparison = 'PrimaryTumor-SolidTissueNormal',

method = 'DESeq2')

deALL <- gdcDEReport(deg = DEGAll, gene.type = 'all', fc = FCfilter, pval = adjpFilter)

deMI <- gdcDEReport(deg = degMI, gene.type = 'all', fc = FCfilter, pval = adjpFilter)

deMIout=cbind(row.names(deMI),deMI)

write.table(deMIout, file='miRNA.diff.txt', sep='\t', quote=F, row.names=F)

allDiff=gdcDEReport(deg = degMI, gene.type = 'all', fc = 0, pval = 1)

Significant=ifelse((allDiff$FDR<adjpFilter & abs(allDiff$logFC)>logFCfilter), ifelse(allDiff$logFC>logFCfilter,"Up","Down"), "Not")

p = ggplot(allDiff, aes(logFC, -log10(FDR)))+

geom_point(aes(col=Significant))+

scale_color_manual(values=c("green", "black", "red"))+

labs(title = " ")+

theme(plot.title = element_text(size = 16, hjust = 0.5, face = "bold"))

p=p+theme_bw()

pdf("miRNA.vol.pdf",width=5.5,height=5)

print(p)

dev.off()

pdf(file="miRNA.heatmap.pdf",width=8,height=6)

degName = rownames(deMI)

gdcHeatmap(deg.id = degName, metadata = metaMatrix.MIR, rna.expr = mirExpr)

dev.off()

deLNC <- gdcDEReport(deg = DEGAll, gene.type = 'long_non_coding', fc = FCfilter, pval = adjpFilter)

write.table(deLNC, file='lncRNA.diff.txt', sep='\t', quote=F, row.names=F)

allDiff=gdcDEReport(deg = DEGAll, gene.type = 'long_non_coding', fc = 0, pval = 1)

Significant=ifelse((allDiff$FDR<adjpFilter & abs(allDiff$logFC)>logFCfilter), ifelse(allDiff$logFC>logFCfilter,"Up","Down"), "Not")

p = ggplot(allDiff, aes(logFC, -log10(FDR)))+

geom_point(aes(col=Significant))+

scale_color_manual(values=c("green", "black", "red"))+

labs(title = " ")+

theme(plot.title = element_text(size = 16, hjust = 0.5, face = "bold"))

p=p+theme_bw()

pdf("lncRNA.vol.pdf",width=5.5,height=5)

print(p)

dev.off()

pdf(file="lncRNA.heatmap.pdf",width=8,height=6)

degName = rownames(deLNC)

gdcHeatmap(deg.id = degName, metadata = metaMatrix.RNA, rna.expr = rnaExpr)

dev.off()

dePC <- gdcDEReport(deg = DEGAll, gene.type = 'protein_coding', fc = FCfilter, pval = adjpFilter)

write.table(dePC, file='mRNA.diff.txt', sep='\t', quote=F, row.names=F)

allDiff=gdcDEReport(deg = DEGAll, gene.type = 'protein_coding', fc = 0, pval = 1)

Significant=ifelse((allDiff$FDR<adjpFilter & abs(allDiff$logFC)>logFCfilter), ifelse(allDiff$logFC>logFCfilter,"Up","Down"), "Not")

p = ggplot(allDiff, aes(logFC, -log10(FDR)))+

geom_point(aes(col=Significant))+

scale_color_manual(values=c("green", "black", "red"))+

labs(title = " ")+

theme(plot.title = element_text(size = 16, hjust = 0.5, face = "bold"))

p=p+theme_bw()

pdf("mRNA.vol.pdf",width=5.5,height=5)

print(p)

dev.off()

pdf(file="mRNA.heatmap.pdf",width=8,height=6)

degName = rownames(dePC)

gdcHeatmap(deg.id = degName, metadata = metaMatrix.RNA, rna.expr = rnaExpr)

dev.off()

#Barplot

pdf("barplot.pdf",width=8,height=8)

gdcBarPlot(deg = deALL, angle = 45, data.type = 'RNAseq')

dev.off()

ceOutput <- gdcCEAnalysis(lnc = rownames(deLNC),

pc = rownames(dePC),

deMIR = rownames(deMI),

lnc.targets = 'starBase', ###'spongeScan', 'starBase', and 'miRcode'

pc.targets = 'starBase', ###'spongeScan', 'starBase', and 'miRcode'

rna.expr = rnaExpr,

mir.expr = mirExpr)

#ceRNA

ceOutput2 <- ceOutput[ceOutput$hyperPValue<hyperPfilter &

ceOutput$corPValue<corPfilter & ceOutput$regSim != 0,]

write.table(ceOutput2, file='ceRNA.score.txt', sep='\t', quote=F, row.names=F) ### Table of Cytoscape

#cytoscape

edges <- gdcExportNetwork(ceNetwork = ceOutput2, net = 'edges')

nodes <- gdcExportNetwork(ceNetwork = ceOutput2, net = 'nodes')

edges=edges[which(edges[,2] %in% rownames(deMI)),]

nodes=nodes[which(nodes[,1] %in% c(as.vector(edges[,1]),rownames(deMI))),]

write.table(edges, file='ceRNA.edges.txt', sep='\t', quote=F, row.names=F) ### Network of Cytoscape

write.table(nodes, file='ceRNA.nodes.txt', sep='\t', quote=F, row.names=F) ### Table of Cytoscape

sameSample=intersect(colnames(rnaExpr),colnames(mirExpr))

rnaExpr=rnaExpr[,sameSample]

mirExpr=mirExpr[,sameSample]

allExp=rbind(rnaExpr,mirExpr)

expOut=allExp[as.vector(nodes[,1]),]

expOut=cbind(Symbol=nodes[,2],expOut)

write.table(expOut, file="networkExp.txt", sep='\t', quote=F, row.names=F)

1. edgeR-GDCRNATools

library("DESeq2")

library("limma")

library("edgeR")

#if (!requireNamespace("BiocManager", quietly = TRUE))

# install.packages("BiocManager")

#BiocManager::install("GDCRNATools")

#install.packages("ggplot2")

library(GDCRNATools)

library(ggplot2)

adjpFilter=0.05

logFCfilter=1

FCfilter=2^logFCfilter

hyperPfilter=0.05

corPfilter=0.05 setwd("C:\\Users\\86186\\Desktop\\11.GDCRNATools")

project <- 'TCGA-HNSC'

rnadir <- paste(project, 'RNAseq', sep='/')

mirdir <- paste(project, 'miRNAs', sep='/')

gdcRNADownload(project.id = project,

data.type = 'RNAseq',

write.manifest = FALSE,

method = 'gdc-client',

directory = rnadir)

gdcRNADownload(project.id = project,

data.type = 'miRNAs',

write.manifest = FALSE,

method = 'gdc-client',

directory = mirdir)

metaMatrix.RNA <- gdcParseMetadata(project.id = project,

data.type = 'RNAseq',

write.meta = FALSE)

metaMatrix.RNA <- gdcFilterDuplicate(metaMatrix.RNA)

metaMatrix.RNA <- gdcFilterSampleType(metaMatrix.RNA)

miRNA metadata

metaMatrix.MIR <- gdcParseMetadata(project.id = project,

data.type = 'miRNAs',

write.meta = FALSE)

#miRNA metadata

metaMatrix.MIR <- gdcFilterDuplicate(metaMatrix.MIR)

metaMatrix.MIR <- gdcFilterSampleType(metaMatrix.MIR)

rnaCounts <- gdcRNAMerge(metadata = metaMatrix.RNA,

path = rnadir,

organized = FALSE, ## if target data are in folders

data.type = 'RNAseq')

mirCounts <- gdcRNAMerge(metadata = metaMatrix.MIR,

path = mirdir,

organized = FALSE, ## if target data are in folders

data.type = 'miRNAs')

rnaExpr <- gdcVoomNormalization(counts = rnaCounts, filter = FALSE)

mirExpr <- gdcVoomNormalization(counts = mirCounts, filter = FALSE)

DEGAll <- gdcDEAnalysis(counts = rnaCounts,

group = metaMatrix.RNA$sample_type,

comparison = 'PrimaryTumor-SolidTissueNormal',

method = 'edgeR')

degMI <- gdcDEAnalysis(counts = mirCounts,

group = metaMatrix.MIR$sample_type,

comparison = 'PrimaryTumor-SolidTissueNormal',

method = 'edgeR')

deALL <- gdcDEReport(deg = DEGAll, gene.type = 'all', fc = FCfilter, pval = adjpFilter)

miRNA

deMI <- gdcDEReport(deg = degMI, gene.type = 'all', fc = FCfilter, pval = adjpFilter)

deMIout=cbind(row.names(deMI),deMI)

write.table(deMIout, file='miRNA.diff.txt', sep='\t', quote=F, row.names=F)

#miRNA

allDiff=gdcDEReport(deg = degMI, gene.type = 'all', fc = 0, pval = 1)

Significant=ifelse((allDiff$FDR<adjpFilter & abs(allDiff$logFC)>logFCfilter), ifelse(allDiff$logFC>logFCfilter,"Up","Down"), "Not")

p = ggplot(allDiff, aes(logFC, -log10(FDR)))+

geom_point(aes(col=Significant))+

scale_color_manual(values=c("green", "black", "red"))+

labs(title = " ")+

theme(plot.title = element_text(size = 16, hjust = 0.5, face = "bold"))

p=p+theme_bw()

pdf("miRNA.vol.pdf",width=5.5,height=5)

print(p)

dev.off()

pdf(file="miRNA.heatmap.pdf",width=8,height=6)

degName = rownames(deMI)

gdcHeatmap(deg.id = degName, metadata = metaMatrix.MIR, rna.expr = mirExpr)

dev.off()

DE-lncRNA

deLNC <- gdcDEReport(deg = DEGAll, gene.type = 'long_non_coding', fc = FCfilter, pval = adjpFilter)

write.table(deLNC, file='lncRNA.diff.txt', sep='\t', quote=F, row.names=F)

lncRNA火山图

allDiff=gdcDEReport(deg = DEGAll, gene.type = 'long_non_coding', fc = 0, pval = 1)

Significant=ifelse((allDiff$FDR<adjpFilter & abs(allDiff$logFC)>logFCfilter), ifelse(allDiff$logFC>logFCfilter,"Up","Down"), "Not")

p = ggplot(allDiff, aes(logFC, -log10(FDR)))+

geom_point(aes(col=Significant))+

scale_color_manual(values=c("green", "black", "red"))+

labs(title = " ")+

theme(plot.title = element_text(size = 16, hjust = 0.5, face = "bold"))

p=p+theme_bw()

pdf("lncRNA.vol.pdf",width=5.5,height=5)

print(p)

dev.off()

#差异lncRNA热图

pdf(file="lncRNA.heatmap.pdf",width=8,height=6)

degName = rownames(deLNC)

gdcHeatmap(deg.id = degName, metadata = metaMatrix.RNA, rna.expr = rnaExpr)

dev.off()

DE-mRNA

dePC <- gdcDEReport(deg = DEGAll, gene.type = 'protein_coding', fc = FCfilter, pval = adjpFilter)

write.table(dePC, file='mRNA.diff.txt', sep='\t', quote=F, row.names=F)

#mRNA火山图

allDiff=gdcDEReport(deg = DEGAll, gene.type = 'protein_coding', fc = 0, pval = 1)

Significant=ifelse((allDiff$FDR<adjpFilter & abs(allDiff$logFC)>logFCfilter), ifelse(allDiff$logFC>logFCfilter,"Up","Down"), "Not")

p = ggplot(allDiff, aes(logFC, -log10(FDR)))+

geom_point(aes(col=Significant))+

scale_color_manual(values=c("green", "black", "red"))+

labs(title = " ")+

theme(plot.title = element_text(size = 16, hjust = 0.5, face = "bold"))

p=p+theme_bw()

pdf("mRNA.vol.pdf",width=5.5,height=5)

print(p)

dev.off()

#DE-mRNA热图

pdf(file="mRNA.heatmap.pdf",width=8,height=6)

degName = rownames(dePC)

gdcHeatmap(deg.id = degName, metadata = metaMatrix.RNA, rna.expr = rnaExpr)

dev.off()

#Barplot

pdf("barplot.pdf",width=8,height=8)

gdcBarPlot(deg = deALL, angle = 45, data.type = 'RNAseq')

dev.off()

###ceRNA

ceOutput <- gdcCEAnalysis(lnc = rownames(deLNC),

pc = rownames(dePC),

deMIR = rownames(deMI),

lnc.targets = 'starBase', ###'spongeScan', 'starBase', and 'miRcode'

pc.targets = 'starBase', ###'spongeScan', 'starBase', and 'miRcode'

rna.expr = rnaExpr,

mir.expr = mirExpr)

#ceRNA filter

ceOutput2 <- ceOutput[ceOutput$hyperPValue<hyperPfilter &

ceOutput$corPValue<corPfilter & ceOutput$regSim != 0,]

write.table(ceOutput2, file='ceRNA.score.txt', sep='\t', quote=F, row.names=F) ### Table of Cytoscape

#cytoscape

edges <- gdcExportNetwork(ceNetwork = ceOutput2, net = 'edges')

nodes <- gdcExportNetwork(ceNetwork = ceOutput2, net = 'nodes')

edges=edges[which(edges[,2] %in% rownames(deMI)),]

nodes=nodes[which(nodes[,1] %in% c(as.vector(edges[,1]),rownames(deMI))),]

write.table(edges, file='ceRNA.edges.txt', sep='\t', quote=F, row.names=F) ### Network of Cytoscape

write.table(nodes, file='ceRNA.nodes.txt', sep='\t', quote=F, row.names=F) ### Table of Cytoscape

sameSample=intersect(colnames(rnaExpr),colnames(mirExpr))

rnaExpr=rnaExpr[,sameSample]

mirExpr=mirExpr[,sameSample]

allExp=rbind(rnaExpr,mirExpr)

expOut=allExp[as.vector(nodes[,1]),]

expOut=cbind(Symbol=nodes[,2],expOut)

write.table(expOut, file="networkExp.txt", sep='\t', quote=F, row.names=F)

1. Limms-GDCRNATools

#if (!requireNamespace("BiocManager", quietly = TRUE))

# install.packages("BiocManager")

#BiocManager::install("GDCRNATools")

#install.packages("ggplot2")

library(GDCRNATools)

library(ggplot2)

adjpFilter=0.05

logFCfilter=1

FCfilter=2^logFCfilter

hyperPfilter=0.05

corPfilter=0.05 setwd("C:\\Users\\86186\\Desktop\\11.GDCRNATools-limma")

project <- 'TCGA-HNSC'

rnadir <- paste(project, 'RNAseq', sep='/')

mirdir <- paste(project, 'miRNAs', sep='/')

gdcRNADownload(project.id = project,

data.type = 'RNAseq',

write.manifest = FALSE,

method = 'gdc-client',

directory = rnadir)

###下载miRNA数据

gdcRNADownload(project.id = project,

data.type = 'miRNAs',

write.manifest = FALSE,

method = 'gdc-client',

directory = mirdir)

###获取转录组metadata

metaMatrix.RNA <- gdcParseMetadata(project.id = project,

data.type = 'RNAseq',

write.meta = FALSE)

#转录组metadata过滤

metaMatrix.RNA <- gdcFilterDuplicate(metaMatrix.RNA)

metaMatrix.RNA <- gdcFilterSampleType(metaMatrix.RNA)

###获取miRNA metadata

metaMatrix.MIR <- gdcParseMetadata(project.id = project,

data.type = 'miRNAs',

write.meta = FALSE)

#miRNA metadata过滤

metaMatrix.MIR <- gdcFilterDuplicate(metaMatrix.MIR)

metaMatrix.MIR <- gdcFilterSampleType(metaMatrix.MIR)

###转录组数据合并

rnaCounts <- gdcRNAMerge(metadata = metaMatrix.RNA,

path = rnadir,

organized = FALSE, ## if target data are in folders

data.type = 'RNAseq')

###miRNA数据合并

mirCounts <- gdcRNAMerge(metadata = metaMatrix.MIR,

path = mirdir,

organized = FALSE, ## if target data are in folders

data.type = 'miRNAs')

###转录组数据矫正

rnaExpr <- gdcVoomNormalization(counts = rnaCounts, filter = FALSE)

###miRNA数据矫正

mirExpr <- gdcVoomNormalization(counts = mirCounts, filter = FALSE)

###转录组差异分析

DEGAll <- gdcDEAnalysis(counts = rnaCounts,

group = metaMatrix.RNA$sample_type,

comparison = 'PrimaryTumor-SolidTissueNormal',

method = 'limma')

###miRNA差异分析

degMI <- gdcDEAnalysis(counts = mirCounts,

group = metaMatrix.MIR$sample_type,

comparison = 'PrimaryTumor-SolidTissueNormal',

method = 'limma')

#所有基因差异

deALL <- gdcDEReport(deg = DEGAll, gene.type = 'all', fc = FCfilter, pval = adjpFilter)

#输出差异的miRNA

deMI <- gdcDEReport(deg = degMI, gene.type = 'all', fc = FCfilter, pval = adjpFilter)

deMIout=cbind(row.names(deMI),deMI)

write.table(deMIout, file='miRNA.diff.txt', sep='\t', quote=F, row.names=F)

#miRNA火山图

allDiff=gdcDEReport(deg = degMI, gene.type = 'all', fc = 0, pval = 1)

Significant=ifelse((allDiff$FDR<adjpFilter & abs(allDiff$logFC)>logFCfilter), ifelse(allDiff$logFC>logFCfilter,"Up","Down"), "Not")

p = ggplot(allDiff, aes(logFC, -log10(FDR)))+

geom_point(aes(col=Significant))+

scale_color_manual(values=c("green", "black", "red"))+

labs(title = " ")+

theme(plot.title = element_text(size = 16, hjust = 0.5, face = "bold"))

p=p+theme_bw()

pdf("miRNA.vol.pdf",width=5.5,height=5)

print(p)

dev.off()

#差异miRNA热图

pdf(file="miRNA.heatmap.pdf",width=8,height=6)

degName = rownames(deMI)

gdcHeatmap(deg.id = degName, metadata = metaMatrix.MIR, rna.expr = mirExpr)

dev.off()

#差异lncRNA

deLNC <- gdcDEReport(deg = DEGAll, gene.type = 'long_non_coding', fc = FCfilter, pval = adjpFilter)

write.table(deLNC, file='lncRNA.diff.txt', sep='\t', quote=F, row.names=F)

#lncRNA火山图

allDiff=gdcDEReport(deg = DEGAll, gene.type = 'long_non_coding', fc = 0, pval = 1)

Significant=ifelse((allDiff$FDR<adjpFilter & abs(allDiff$logFC)>logFCfilter), ifelse(allDiff$logFC>logFCfilter,"Up","Down"), "Not")

p = ggplot(allDiff, aes(logFC, -log10(FDR)))+

geom_point(aes(col=Significant))+

scale_color_manual(values=c("green", "black", "red"))+

labs(title = " ")+

theme(plot.title = element_text(size = 16, hjust = 0.5, face = "bold"))

p=p+theme_bw()

pdf("lncRNA.vol.pdf",width=5.5,height=5)

print(p)

dev.off()

#差异lncRNA热图

pdf(file="lncRNA.heatmap.pdf",width=8,height=6)

degName = rownames(deLNC)

gdcHeatmap(deg.id = degName, metadata = metaMatrix.RNA, rna.expr = rnaExpr)

dev.off()

#差异mRNA

dePC <- gdcDEReport(deg = DEGAll, gene.type = 'protein_coding', fc = FCfilter, pval = adjpFilter)

write.table(dePC, file='mRNA.diff.txt', sep='\t', quote=F, row.names=F)

#mRNA火山图

allDiff=gdcDEReport(deg = DEGAll, gene.type = 'protein_coding', fc = 0, pval = 1)

Significant=ifelse((allDiff$FDR<adjpFilter & abs(allDiff$logFC)>logFCfilter), ifelse(allDiff$logFC>logFCfilter,"Up","Down"), "Not")

p = ggplot(allDiff, aes(logFC, -log10(FDR)))+

geom_point(aes(col=Significant))+

scale_color_manual(values=c("green", "black", "red"))+

labs(title = " ")+

theme(plot.title = element_text(size = 16, hjust = 0.5, face = "bold"))

p=p+theme_bw()

pdf("mRNA.vol.pdf",width=5.5,height=5)

print(p)

dev.off()

#差异mRNA热图

pdf(file="mRNA.heatmap.pdf",width=8,height=6)

degName = rownames(dePC)

gdcHeatmap(deg.id = degName, metadata = metaMatrix.RNA, rna.expr = rnaExpr)

dev.off()

#Barplot

pdf("barplot.pdf",width=8,height=8)

gdcBarPlot(deg = deALL, angle = 45, data.type = 'RNAseq')

dev.off()

###ceRNA网络关系

ceOutput <- gdcCEAnalysis(lnc = rownames(deLNC),

pc = rownames(dePC),

deMIR = rownames(deMI),

lnc.targets = 'starBase', ###'spongeScan', 'starBase', and 'miRcode'

pc.targets = 'starBase', ###'spongeScan', 'starBase', and 'miRcode'

rna.expr = rnaExpr,

mir.expr = mirExpr)

#ceRNA网络过滤

ceOutput2 <- ceOutput[ceOutput$hyperPValue<hyperPfilter &

ceOutput$corPValue<corPfilter & ceOutput$regSim != 0,]

write.table(ceOutput2, file='ceRNA.score.txt', sep='\t', quote=F, row.names=F) ### Table of Cytoscape

#cytoscape需要的输入文件

edges <- gdcExportNetwork(ceNetwork = ceOutput2, net = 'edges')

nodes <- gdcExportNetwork(ceNetwork = ceOutput2, net = 'nodes')

edges=edges[which(edges[,2] %in% rownames(deMI)),]

nodes=nodes[which(nodes[,1] %in% c(as.vector(edges[,1]),rownames(deMI))),]

write.table(edges, file='ceRNA.edges.txt', sep='\t', quote=F, row.names=F) ### Network of Cytoscape

write.table(nodes, file='ceRNA.nodes.txt', sep='\t', quote=F, row.names=F) ### Table of Cytoscape

#输出ceRNA各个节点表达量

sameSample=intersect(colnames(rnaExpr),colnames(mirExpr))

rnaExpr=rnaExpr[,sameSample]

mirExpr=mirExpr[,sameSample]

allExp=rbind(rnaExpr,mirExpr)

expOut=allExp[as.vector(nodes[,1]),]

expOut=cbind(Symbol=nodes[,2],expOut)

write.table(expOut, file="networkExp.txt", sep='\t', quote=F, row.names=F)

1. mergeTime

#if (!requireNamespace("BiocManager", quietly = TRUE))

# install.packages("BiocManager")

#BiocManager::install("limma")

library(limma)

expFile="networkExp.txt"

cliFile="time.txt" setwd("C:\\Users\\86186\\Desktop\\HNSCC-CeRNA\\15.mergeTime")

rt=read.table(expFile,sep="\t",header=T,check.names=F)

rt=as.matrix(rt)

rownames(rt)=rt[,1]

exp=rt[,2:ncol(rt)]

dimnames=list(rownames(exp),colnames(exp))

data=matrix(as.numeric(as.matrix(exp)),nrow=nrow(exp),dimnames=dimnames)

data=avereps(data)

data=data[rowMeans(data)>0,]

group=sapply(strsplit(colnames(data),"\\-"),"[",4)

group=sapply(strsplit(group,""),"[",1)

group=gsub("2","1",group)

data=data[,group==0]

colnames(data)=gsub("(.*?)\\-(.*?)\\-(.*?)\\-.*","\\1\\-\\2\\-\\3",colnames(data))

data=t(data)

data=avereps(data)

cli=read.table(cliFile,sep="\t",check.names=F,header=T,row.names=1)

sameSample=intersect(row.names(data),row.names(cli))

data=data[sameSample,]

cli=cli[sameSample,]

out=cbind(cli,data)

out=cbind(id=row.names(out),out)

write.table(out,file="expTime.txt",sep="\t",row.names=F,quote=F)

1. geneCox

#install.packages('survival')

#install.packages('survminer')

#install.packages("glmnet")

library(survival)

library(survminer)

library(glmnet)

coxPfilter=0.05 setwd("C:\\Users\\86186\\Desktop\\HNSCC-CeRNA\\17.geneCox") rt=read.table("expTime.txt",header=T,sep="\t",check.names=F,row.names=1) rt$futime=rt$futime/365

outTab=data.frame()

sigGenes=c("futime","fustat")

for(i in colnames(rt[,3:ncol(rt)])){

if(sd(rt[,i])<0.001){next}

cox <- coxph(Surv(futime, fustat) ~ rt[,i], data = rt)

coxSummary = summary(cox)

coxP=coxSummary$coefficients[,"Pr(>|z|)"]

if(coxP<coxPfilter){

sigGenes=c(sigGenes,i)

outTab=rbind(outTab,

cbind(id=i,

HR=coxSummary$conf.int[,"exp(coef)"],

HR.95L=coxSummary$conf.int[,"lower .95"],

HR.95H=coxSummary$conf.int[,"upper .95"],

pvalue=coxSummary$coefficients[,"Pr(>|z|)"])

)

}

}

write.table(outTab,file="uni.Cox.txt",sep="\t",row.names=F,quote=F)

uniSigExp=rt[,sigGenes]

uniSigExp=cbind(id=row.names(uniSigExp),uniSigExp)

write.table(uniSigExp,file="uni.SigExp.txt",sep="\t",row.names=F,quote=F)

###lasso筛选基因

rt=read.table("uni.SigExp.txt",header=T,sep="\t",row.names=1,check.names=F)

rt$futime[rt$futime<=0]=0.003

x=as.matrix(rt[,c(3:ncol(rt))])

y=data.matrix(Surv(rt$futime,rt$fustat))

fit <- glmnet(x, y, family = "cox", maxit = 1000)

pdf("lasso.lambda.pdf")

plot(fit, xvar = "lambda", label = TRUE)

dev.off()

cvfit <- cv.glmnet(x, y, family="cox", maxit = 1000)

pdf("lasso.cvfit.pdf")

plot(cvfit)

abline(v=log(c(cvfit$lambda.min,cvfit$lambda.1se)),lty="dashed")

dev.off()

coef <- coef(fit, s = cvfit$lambda.min)

index <- which(coef != 0)

actCoef <- coef[index]

lassoGene=row.names(coef)[index]

lassoGene=c("futime","fustat",lassoGene)

lassoSigExp=rt[,lassoGene]

lassoSigExp=cbind(id=row.names(lassoSigExp),lassoSigExp)

write.table(lassoSigExp,file="lasso.SigExp.txt",sep="\t",row.names=F,quote=F)

#COX模型构建

rt=read.table("lasso.SigExp.txt",header=T,sep="\t",check.names=F,row.names=1)

multiCox=coxph(Surv(futime, fustat) ~ ., data = rt)

#multiCox=step(multiCox,direction = "both") #多因素cox根据AIC值进行过滤，如果剩余的基因数目多，可以把这行前面的#号去掉

multiCoxSum=summary(multiCox)

outTab=data.frame()

outTab=cbind(

coef=multiCoxSum$coefficients[,"coef"],

HR=multiCoxSum$conf.int[,"exp(coef)"],

HR.95L=multiCoxSum$conf.int[,"lower .95"],

HR.95H=multiCoxSum$conf.int[,"upper .95"],

pvalue=multiCoxSum$coefficients[,"Pr(>|z|)"])

outTab=cbind(id=row.names(outTab),outTab)

outTab=gsub("`","",outTab)

write.table(outTab,file="multi.Cox.txt",sep="\t",row.names=F,quote=F)

riskScore=predict(multiCox,type="risk",newdata=rt)

coxGene=rownames(multiCoxSum$coefficients)

coxGene=gsub("`","",coxGene)

outCol=c("futime","fustat",coxGene)

risk=as.vector(ifelse(riskScore>median(riskScore),"high","low"))

riskOut=cbind(rt[,outCol],riskScore,risk)

riskOut=cbind(id=rownames(riskOut),riskOut)

write.table(riskOut,file="geneRisk.txt",sep="\t",quote=F,row.names=F)

pdf(file="multi.forest.pdf",width = 10,height = 6,onefile = FALSE)

ggforest(multiCox,

main = "Hazard ratio",

cpositions = c(0.02,0.22, 0.4),

fontsize = 0.7,

refLabel = "reference",

noDigits = 2)

dev.off()

1. geneNomo

#install.packages("rms")

library(rms)

setwd("C:\\Users\\86186\\Desktop\\HNSCC-CeRNA\\19.geneNomo") #TCGA列线图绘制

riskFile="geneRisk.txt"

outFile="Nomogram.pdf" risk=read.table(riskFile,header=T,sep="\t",check.names=F,row.names=1) rt=risk[,1:(ncol(risk)-2)]

#数据打包

dd <- datadist(rt)

options(datadist="dd")

f <- cph(Surv(futime, fustat) ~ANLN+CFL2+ITGA5+KDELC1+KIF23+NFIA+PTX3+RELT+TMC7, x=T, y=T, surv=T, data=rt, time.inc=1)

surv <- Survival(f)

#建立nomogram

nom <- nomogram(f, fun=list(function(x) surv(1, x), function(x) surv(2, x), function(x) surv(3, x)),

lp=F, funlabel=c("1-year survival", "2-year survival", "3-year survival"),

maxscale=100,

fun.at=c(0.99, 0.9, 0.8, 0.7, 0.5, 0.3,0.1,0.01))

#nomogram可视化

pdf(file=outFile,height=6,width=9)

plot(nom)

dev.off()

#calibration curve

time=3 calibration

f <- cph(Surv(futime, fustat) ~ANLN+CFL2+ITGA5+KDELC1+KIF23+NFIA+PTX3+RELT+TMC7, x=T, y=T, surv=T, data=rt, time.inc=time)

cal <- calibrate(f, cmethod="KM", method="boot", u=time, m=100, B=1000)

pdf(file="calibration.pdf",height=6,width=8)

plot(cal,xlab="Nomogram-Predicted Probability of 3-Year OS",ylab="Actual 3-Year OS(proportion)",col="red",sub=F)

dev.off()

1. CIBERSORT

#' CIBERSORT R script v1.03

#' Note: Signature matrix construction is not currently available; use java version for full functionality.

#' Author: Aaron M. Newman, Stanford University (amnewman@stanford.edu)

#' Requirements:

#' R v3.0 or later. (dependencies below might not work properly with earlier versions)

#' install.packages('e1071')

#' install.pacakges('parallel')

#' install.packages('preprocessCore')

#' if preprocessCore is not available in the repositories you have selected, run the following:

#' source("http://bioconductor.org/biocLite.R")

#' biocLite("preprocessCore")

#' Windows users using the R GUI may need to Run as Administrator to install or update packages.

#' This script uses 3 parallel processes. Since Windows does not support forking, this script will run

#' single-threaded in Windows.

#'

#' Usage:

#' Navigate to directory containing R script

#'

#' In R:

#' source('CIBERSORT.R')

#' results <- CIBERSORT('sig_matrix_file.txt','mixture_file.txt', perm, QN)

#'

#' Options:

#' i) perm = No. permutations; set to >=100 to calculate p-values (default = 0)

#' ii) QN = Quantile normalization of input mixture (default = TRUE)

#'

#' Input: signature matrix and mixture file, formatted as specified at http://cibersort.stanford.edu/tutorial.php

#' Output: matrix object containing all results and tabular data written to disk 'CIBERSORT-Results.txt'

#' License: http://cibersort.stanford.edu/CIBERSORT_License.txt

#' Core algorithm

#' @param X cell-specific gene expression

#' @param y mixed expression per sample

#' @export

CoreAlg <- function(X, y){

#try different values of nu

svn_itor <- 3

res <- function(i){

if(i==1){nus <- 0.25}

if(i==2){nus <- 0.5}

if(i==3){nus <- 0.75}

model<-svm(X,y,type="nu-regression",kernel="linear",nu=nus,scale=F)

model

}

if(Sys.info()['sysname'] == 'Windows') out <- mclapply(1:svn_itor, res, mc.cores=1) else

out <- mclapply(1:svn_itor, res, mc.cores=svn_itor)

nusvm <- rep(0,svn_itor)

corrv <- rep(0,svn_itor)

#do cibersort

t <- 1

while(t <= svn_itor) {

weights = t(out[[t]]$coefs) %*% out[[t]]$SV

weights[which(weights<0)]<-0

w<-weights/sum(weights)

u <- sweep(X,MARGIN=2,w,'*')

k <- apply(u, 1, sum)

nusvm[t] <- sqrt((mean((k - y)^2)))

corrv[t] <- cor(k, y)

t <- t + 1

}

#pick best model

rmses <- nusvm

mn <- which.min(rmses)

model <- out[[mn]]

#get and normalize coefficients

q <- t(model$coefs) %*% model$SV

q[which(q<0)]<-0

w <- (q/sum(q))

mix_rmse <- rmses[mn]

mix_r <- corrv[mn]

newList <- list("w" = w, "mix_rmse" = mix_rmse, "mix_r" = mix_r)

}

#' do permutations

#' @param perm Number of permutations

#' @param X cell-specific gene expression

#' @param y mixed expression per sample

#' @export

doPerm <- function(perm, X, Y){

itor <- 1

Ylist <- as.list(data.matrix(Y))

dist <- matrix()

while(itor <= perm){

#print(itor)

#random mixture

yr <- as.numeric(Ylist[sample(length(Ylist),dim(X)[1])])

#standardize mixture

yr <- (yr - mean(yr)) / sd(yr)

#run CIBERSORT core algorithm

result <- CoreAlg(X, yr)

mix_r <- result$mix_r

#store correlation

if(itor == 1) {dist <- mix_r}

else {dist <- rbind(dist, mix_r)}

itor <- itor + 1

}

newList <- list("dist" = dist)

}

#' Main functions

#' @param sig_matrix file path to gene expression from isolated cells

#' @param mixture_file heterogenous mixed expression

#' @param perm Number of permutations

#' @param QN Perform quantile normalization or not (TRUE/FALSE)

#' @export

CIBERSORT <- function(sig_matrix, mixture_file, perm=0, QN=TRUE){

library(e1071)

library(parallel)

library(preprocessCore)

#read in data

X <- read.table(sig_matrix,header=T,sep="\t",row.names=1,check.names=F)

Y <- read.table(mixture_file, header=T, sep="\t", row.names=1,check.names=F)

X <- data.matrix(X)

Y <- data.matrix(Y)

#order

X <- X[order(rownames(X)),]

Y <- Y[order(rownames(Y)),]

P <- perm #number of permutations

#anti-log if max < 50 in mixture file

if(max(Y) < 50) {Y <- 2^Y}

#quantile normalization of mixture file

if(QN == TRUE){

tmpc <- colnames(Y)

tmpr <- rownames(Y)

Y <- normalize.quantiles(Y)

colnames(Y) <- tmpc

rownames(Y) <- tmpr

}

#intersect genes

Xgns <- row.names(X)

Ygns <- row.names(Y)

YintX <- Ygns %in% Xgns

Y <- Y[YintX,]

XintY <- Xgns %in% row.names(Y)

X <- X[XintY,]

#standardize sig matrix

X <- (X - mean(X)) / sd(as.vector(X))

#empirical null distribution of correlation coefficients

if(P > 0) {nulldist <- sort(doPerm(P, X, Y)$dist)}

#print(nulldist)

header <- c('Mixture',colnames(X),"P-value","Correlation","RMSE")

#print(header)

output <- matrix()

itor <- 1

mixtures <- dim(Y)[2]

pval <- 9999

#iterate through mixtures

while(itor <= mixtures){

y <- Y[,itor]

#standardize mixture

y <- (y - mean(y)) / sd(y)

#run SVR core algorithm

result <- CoreAlg(X, y)

#get results

w <- result$w

mix_r <- result$mix_r

mix_rmse <- result$mix_rmse

#calculate p-value

if(P > 0) {pval <- 1 - (which.min(abs(nulldist - mix_r)) / length(nulldist))}

#print output

out <- c(colnames(Y)[itor],w,pval,mix_r,mix_rmse)

if(itor == 1) {output <- out}

else {output <- rbind(output, out)}

itor <- itor + 1

}

#save results

write.table(rbind(header,output), file="CIBERSORT-Results.txt", sep="\t", row.names=F, col.names=F, quote=F)

#return matrix object containing all results

obj <- rbind(header,output)

obj <- obj[,-1]

obj <- obj[-1,]

obj <- matrix(as.numeric(unlist(obj)),nrow=nrow(obj))

rownames(obj) <- colnames(Y)

colnames(obj) <- c(colnames(X),"P-value","Correlation","RMSE")

obj

}

1. immCeRNA20.run

#install.packages('e1071')

#if (!requireNamespace("BiocManager", quietly = TRUE))

# install.packages("BiocManager")

#BiocManager::install("preprocessCore")

#if (!requireNamespace("BiocManager", quietly = TRUE))

# install.packages("BiocManager")

#BiocManager::install("limma")

library("limma")

expFile="symbol.txt" setwd("C:\\Users\\86186\\Desktop\\HNSCC-CeRNA\\20.CIBERSORT")

rt=read.table(expFile,sep="\t",header=T,check.names=F)

rt=as.matrix(rt)

rownames(rt)=rt[,1]

exp=rt[,2:ncol(rt)]

dimnames=list(rownames(exp),colnames(exp))

data=matrix(as.numeric(as.matrix(exp)),nrow=nrow(exp),dimnames=dimnames)

data=avereps(data)

data=data[rowMeans(data)>0,]

v <-voom(data, plot = F, save.plot = F)

out=v$E

out=rbind(ID=colnames(out),out)

write.table(out,file="uniq.symbol.txt",sep="\t",quote=F,col.names=F) #运行CIBERSORT，

source("immCeRNA20.CIBERSORT.R")

results=CIBERSORT("ref.txt", "uniq.symbol.txt", perm=100, QN=TRUE)

1. immuneCox

#install.packages('survival')

#install.packages('survminer')

#install.packages("glmnet")

library(survival)

library(survminer)

library(glmnet)

coxPfilter=0.05 setwd("C:\\Users\\86186\\Desktop\\HNSCC-CeRNA\\27.immuneCox") rt=read.table("immuneTime.txt",header=T,sep="\t",check.names=F,row.names=1) rt$futime=rt$futime/365

#单因素cox分析

outTab=data.frame()

sigGenes=c("futime","fustat")

for(i in colnames(rt[,3:ncol(rt)])){

if(sd(rt[,i])<0.001){next}

#cox分析

cox <- coxph(Surv(futime, fustat) ~ rt[,i], data = rt)

coxSummary = summary(cox)

coxP=coxSummary$coefficients[,"Pr(>|z|)"]

#保留显著性免疫细胞

if(coxP<coxPfilter){

sigGenes=c(sigGenes,i)

outTab=rbind(outTab,

cbind(id=i,

HR=coxSummary$conf.int[,"exp(coef)"],

HR.95L=coxSummary$conf.int[,"lower .95"],

HR.95H=coxSummary$conf.int[,"upper .95"],

pvalue=coxSummary$coefficients[,"Pr(>|z|)"])

)

}

}

write.table(outTab,file="uni.Cox.txt",sep="\t",row.names=F,quote=F)

uniSigExp=rt[,sigGenes]

uniSigExp=cbind(id=row.names(uniSigExp),uniSigExp)

write.table(uniSigExp,file="uni.SigExp.txt",sep="\t",row.names=F,quote=F)

###lasso筛选免疫细胞

rt=read.table("uni.SigExp.txt",header=T,sep="\t",row.names=1,check.names=F) #读取文件

rt$futime[rt$futime<=0]=0.003

x=as.matrix(rt[,c(3:ncol(rt))])

y=data.matrix(Surv(rt$futime,rt$fustat))

fit <- glmnet(x, y, family = "cox", maxit = 1000)

pdf("lasso.lambda.pdf")

plot(fit, xvar = "lambda", label = TRUE)

dev.off()

cvfit <- cv.glmnet(x, y, family="cox", maxit = 1000)

pdf("lasso.cvfit.pdf")

plot(cvfit)

abline(v=log(c(cvfit$lambda.min,cvfit$lambda.1se)),lty="dashed")

dev.off()

coef <- coef(fit, s = cvfit$lambda.min)

index <- which(coef != 0)

actCoef <- coef[index]

lassoGene=row.names(coef)[index]

lassoGene=c("futime","fustat",lassoGene)

lassoSigExp=rt[,lassoGene]

lassoSigExp=cbind(id=row.names(lassoSigExp),lassoSigExp)

write.table(lassoSigExp,file="lasso.SigExp.txt",sep="\t",row.names=F,quote=F)

#COX模型构建

rt=read.table("lasso.SigExp.txt",header=T,sep="\t",check.names=F,row.names=1)

multiCox=coxph(Surv(futime, fustat) ~ ., data = rt)

multiCox=step(multiCox,direction = "both") #多因素cox根据AIC值进行过滤，如果剩余的免疫细胞数目多，可以把这行前面的#号去掉

multiCoxSum=summary(multiCox)

#输出模型参数

outTab=data.frame()

outTab=cbind(

coef=multiCoxSum$coefficients[,"coef"],

HR=multiCoxSum$conf.int[,"exp(coef)"],

HR.95L=multiCoxSum$conf.int[,"lower .95"],

HR.95H=multiCoxSum$conf.int[,"upper .95"],

pvalue=multiCoxSum$coefficients[,"Pr(>|z|)"])

outTab=cbind(id=row.names(outTab),outTab)

outTab=gsub("`","",outTab)

write.table(outTab,file="multi.Cox.txt",sep="\t",row.names=F,quote=F)

riskScore=predict(multiCox,type="risk",newdata=rt)

coxGene=rownames(multiCoxSum$coefficients)

coxGene=gsub("`","",coxGene)

outCol=c("futime","fustat",coxGene)

risk=as.vector(ifelse(riskScore>median(riskScore),"high","low"))

riskOut=cbind(rt[,outCol],riskScore,risk)

riskOut=cbind(id=rownames(riskOut),riskOut)

write.table(riskOut,file="immuneRisk.txt",sep="\t",quote=F,row.names=F)

pdf(file="multi.forest.pdf",width = 10,height = 6,onefile = FALSE)

ggforest(multiCox,

main = "Hazard ratio",

cpositions = c(0.02,0.22, 0.4),

fontsize = 0.7,

refLabel = "reference",

noDigits = 2)

dev.off()

1. immuneNomo

#install.packages("rms")

library(rms)

setwd("C:\\Users\\86186\\Desktop\\HNSCC-CeRNA\\29.immuneNomo")

#TCGA

riskFile="immuneRisk.txt"

outFile="Nomogram.pdf" risk=read.table(riskFile,header=T,sep="\t",check.names=F,row.names=1)

rt=risk[,1:(ncol(risk)-2)]

dd <- datadist(rt)

options(datadist="dd")

f <- cph(Surv(futime, fustat) ~B_cells_naive+Tregs+Neutrophils, x=T, y=T, surv=T, data=rt, time.inc=1)

surv <- Survival(f)

nomogram

nom <- nomogram(f, fun=list(function(x) surv(1, x), function(x) surv(2, x), function(x) surv(3, x)),

lp=F, funlabel=c("1-year survival", "2-year survival", "3-year survival"),

maxscale=100,

fun.at=c(0.99, 0.9, 0.8, 0.7, 0.5, 0.3,0.1,0.01))

#nomogram可视化

pdf(file=outFile,height=6,width=9)

plot(nom)

dev.off()

#calibration curve

time=3 calibration

f <- cph(Surv(futime, fustat) ~B_cells_naive+Tregs+Neutrophils, x=T, y=T, surv=T, data=rt, time.inc=time)

cal <- calibrate(f, cmethod="KM", method="boot", u=time, m=80, B=1000)

pdf(file="calibration.pdf",height=6,width=8)

plot(cal,xlab="Nomogram-Predicted Probability of 3-Year OS",ylab="Actual 3-Year OS(proportion)",col="red",sub=F)

dev.off()

1. Corrplot

#install.packages("corrplot")

library(corrplot)

geneRiskFile="geneRisk.txt" immuneRiskFile="immuneRisk.txt" setwd("C:\\Users\\86186\\Desktop\\HNSCC-CeRNA\\31.corrplot")

geneRisk=read.table(geneRiskFile,header=T,sep="\t",check.names=F,row.names=1)

geneRisk=geneRisk[,3:(ncol(geneRisk)-2)]

immuneRisk=read.table(immuneRiskFile,header=T,sep="\t",check.names=F,row.names=1)

immuneRisk=immuneRisk[,3:(ncol(immuneRisk)-2)]

sameSample=intersect(row.names(geneRisk),row.names(immuneRisk))

geneRisk=geneRisk[sameSample,]

immuneRisk=immuneRisk[sameSample,]

data=cbind(geneRisk,immuneRisk)

pdf("corrplot.pdf",height=12,width=12) par(oma=c(0.5,0.5,0.5,1.2))

data=data[,colMeans(data)>0]

M=cor(data)

corrplot(M,

order="original",

method = "color",

addCoef.col = "black",

diag = TRUE,

tl.col="black",

col=colorRampPalette(c("blue", "white", "red"))(50))

dev.off()

1. indep analysis

#install.packages('survival')

library(survival)

setwd("C:\\Users\\86186\\Desktop\\20.Indep")

rt=read.table("indepInput.txt",header=T,sep="\t",check.names=F,row.names=1)

uniTab=data.frame()

for(i in colnames(rt[,3:ncol(rt)])){

cox <- coxph(Surv(futime, fustat) ~ rt[,i], data = rt)

coxSummary = summary(cox)

uniTab=rbind(uniTab,

cbind(id=i,

B=coxSummary$coefficients[,"coef"],

SE=coxSummary$coefficients[,"se(coef)"],

z=coxSummary$coefficients[,"z"],

HR=coxSummary$conf.int[,"exp(coef)"],

HR.95L=coxSummary$conf.int[,"lower .95"],

HR.95H=coxSummary$conf.int[,"upper .95"],

pvalue=coxSummary$coefficients[,"Pr(>|z|)"])

)

}

write.table(uniTab,file="uniCox.txt",sep="\t",row.names=F,quote=F)

#多因素独立预后分析

multiCox=coxph(Surv(futime, fustat) ~ ., data = rt)

multiCoxSum=summary(multiCox)

multiTab=data.frame()

multiTab=cbind(

B=multiCoxSum$coefficients[,"coef"],

SE=multiCoxSum$coefficients[,"se(coef)"],

z=multiCoxSum$coefficients[,"z"],

HR=multiCoxSum$conf.int[,"exp(coef)"],

HR.95L=multiCoxSum$conf.int[,"lower .95"],

HR.95H=multiCoxSum$conf.int[,"upper .95"],

pvalue=multiCoxSum$coefficients[,"Pr(>|z|)"])

multiTab=cbind(id=row.names(multiTab),multiTab)

write.table(multiTab,file="multiCox.txt",sep="\t",row.names=F,quote=F)

############绘制森林图函数############

bioForest=function(coxFile=null,forestCol=null,forestFile=null){

#读取输入文件

rt <- read.table(coxFile,header=T,sep="\t",row.names=1,check.names=F)

gene <- rownames(rt)

hr <- sprintf("%.3f",rt$"HR")

hrLow <- sprintf("%.3f",rt$"HR.95L")

hrHigh <- sprintf("%.3f",rt$"HR.95H")

Hazard.ratio <- paste0(hr,"(",hrLow,"-",hrHigh,")")

pVal <- ifelse(rt$pvalue<0.001, "<0.001", sprintf("%.3f", rt$pvalue))

pdf(file=forestFile, width = 6,height = 4.2)

n <- nrow(rt)

nRow <- n+1

ylim <- c(1,nRow)

layout(matrix(c(1,2),nc=2),width=c(3,2.5))

xlim = c(0,3)

par(mar=c(4,2.5,2,1))

plot(1,xlim=xlim,ylim=ylim,type="n",axes=F,xlab="",ylab="")

text.cex=0.8

text(0,n:1,gene,adj=0,cex=text.cex)

text(1.5-0.5*0.2,n:1,pVal,adj=1,cex=text.cex);text(1.5-0.5*0.2,n+1,'pvalue',cex=text.cex,font=2,adj=1)

text(3,n:1,Hazard.ratio,adj=1,cex=text.cex);text(3,n+1,'Hazard ratio',cex=text.cex,font=2,adj=1,)

par(mar=c(4,1,2,1),mgp=c(2,0.5,0))

xlim = c(0,max(as.numeric(hrLow),as.numeric(hrHigh)))

plot(1,xlim=xlim,ylim=ylim,type="n",axes=F,ylab="",xaxs="i",xlab="Hazard ratio")

arrows(as.numeric(hrLow),n:1,as.numeric(hrHigh),n:1,angle=90,code=3,length=0.05,col="darkblue",lwd=2.5)

abline(v=1,col="black",lty=2,lwd=2)

boxcolor = ifelse(as.numeric(hr) > 1, forestCol, forestCol)

points(as.numeric(hr), n:1, pch = 15, col = boxcolor, cex=1.3)

axis(1)

dev.off()

}

bioForest(coxFile="uniCox.txt", forestCol="blue", forestFile="uniForest.pdf")

bioForest(coxFile="multiCox.txt", forestCol="red", forestFile="multiForest.pdf")
